# Bone marrow stromal antigen 2 expressed in cancer cells promotes mammary tumor growth and metastasis

**DOI:** 10.1186/s13058-014-0493-8

**Published:** 2014-12-13

**Authors:** Wadie D Mahauad-Fernandez, Kris A DeMali, Alicia K Olivier, Chioma M Okeoma

**Affiliations:** 1 Department of Microbiology, Carver College of Medicine, University of Iowa, 51 Newton Road, Iowa City, 52242-1109 IA USA; 2 Interdisciplinary Graduate Program in Molecular and Cellular Biology (MCB), University of Iowa, 500 Newton Road, Iowa City, 52242-1109 IA USA; 3 Department of Biochemistry, Carver College of Medicine, University of Iowa, 51 Newton Road, Iowa City, 52242-1109 IA USA; 4 Department of Pathology, Carver College of Medicine, University of Iowa, 51 Newton Road, Iowa City, 52242-1109 IA USA; 5 Department of Pathobiology and Population Medicine, College of Veterinary Medicine, Mississippi State University, 240 Wise Center Drive, Starkville, 39762-6100 MS, USA

## Abstract

**Introduction:**

Several innate immunity genes are overexpressed in human cancers and their roles remain controversial. Bone marrow stromal antigen 2 (BST-2) is one such gene whose role in cancer is not clear. BST-2 is a unique innate immunity gene with both antiviral and pro-tumor functions and therefore can serve as a paradigm for understanding the roles of other innate immunity genes in cancers.

**Methods:**

Meta-analysis of tumors from breast cancer patients obtained from the Gene Expression Omnibus (GEO) and The Cancer Genome Atlas (TCGA) datasets were evaluated for levels of BST-2 expression and for tumor aggressiveness. *In vivo*, we examined the effect of knockdown of BST-2 in two different murine carcinoma cells on tumor growth, metastasis, and survival*. In vitro*, we assessed the effect of carcinoma cell BST-2 knockdown and/or overexpression on adhesion, anchorage-independent growth, migration, and invasion.

**Results:**

BST-2 in breast tumors and mammary cancer cells is a strong predictor of tumor size, tumor aggressiveness, and host survival. In humans, BST-2 mRNA is elevated in metastatic and invasive breast tumors. In mice, orthotopic implantation of mammary tumor cells lacking BST-2 increased tumor latency, decreased primary tumor growth, reduced metastases to distal organs, and prolonged host survival. Furthermore, we found that the cellular basis for the role of BST-2 in promoting tumorigenesis include BST-2-directed enhancement in cancer cell adhesion, anchorage-independency, migration, and invasion.

**Conclusions:**

BST-2 contributes to the emergence of neoplasia and malignant progression of breast cancer. Thus, BST-2 may (1) serve as a biomarker for aggressive breast cancers, and (2) be a novel target for breast cancer therapeutics.

**Electronic supplementary material:**

The online version of this article (doi:10.1186/s13058-014-0493-8) contains supplementary material, which is available to authorized users.

## Introduction

The oncogenesis of breast cancer involves multiple events, including genetic and epigenetic alterations in the behavior of normal and malignant cells, as well as other cells that interact with cancer cells [1]. Such alterations modulate the functions of key host genes, which in turn affect cancer cell behavior including self-sufficiency in growth signals, adhesion, invasion, motility, and survival. Our understanding of specific genes linked to the development and progression of mammary cancer is unraveling. These genes have enabled the development of targeted therapeutics against mammary cancers that are dependent on such genes. However, the goal of eliminating breast cancer has not been met partially because not all cancer driver genes have been identified. In particular, it is not clear how overexpression of innate immunity genes in cancer cells endow these cells tumorigenic potential.

Innate immunity is crucial to host defense. However, some innate immunity genes play paradoxical roles as they prevent [2] and/or promote [3] cancer through mechanisms that are not well defined. It has been shown that the innate immunity gene called bone marrow stromal antigen 2 (BST-2), also known as tetherin, CD317, and HM1.24 is overexpressed in several cancers [4]-[11]. BST-2 is an interferon-inducible type II transmembrane protein that functions as a potent nuclear factor kappa binding (NF-кB) activator [12]. BST-2-mediated NF-кB activation occurs through the YXY motif on the cytoplasmic domain of BST-2 and interaction with TAK1 is required [13],[14]. The activation of NF-кB by BST-2 results in increased production of immune-inflammatory mediators that may inhibit viral replication [13], but may also promote tumorigenesis. In addition to the NF-кB-regulating role, BST-2 is reputed for its tethering and antiviral functions, as its overexpression tethers/retains nascent virions on the surface of infected cells and prevents infection of new target cells [15]-[17]. The tetherin function of BST-2 has been shown to be involved in cell to cell interactions because BST-2 mediates the adhesion of monocytes to endothelial cells [18]; a function that could promote intravasation of immune cells.

Although overexpression of BST-2 tethers virions on the cell membrane and negatively regulates virus replication, it is likely that elevated BST-2 expression might positively influence cancer cell behavior [6],[7],[9],[10],[19]. It has been suggested that increased cancer cell adhesion and resistance to apoptosis *in vitro* is linked to BST-2 expression [18],[20],[21]. However, the functional consequence of BST-2 expression in tumor tissues and cells is completely unknown and there has been no direct demonstration of the involvement of BST-2 in breast tumorigenesis.

Given the role of BST-2 in innate immunity - including its role in NF-кB activation and subsequent transcription of NF-кB-dependent genes, as well as the presence of high levels of BST-2 in breast tumors [21], we hypothesized that BST-2 may promote mammary tumorigenesis. Here, we studied the clinical consequences of BST-2 expression in breast tumors, the functional role of BST-2 in mammary tumorigenesis, and the cellular basis for BST-2-mediated effect on mammary tumorigenesis.

## Methods

### Cell lines

E0771 (a medullary breast adenocarcinoma cell line from C57BL/6 mouse strain) was purchased from CH3 BioSystems (Amherst, NY, USA). 4T1 (a mouse mammary carcinoma cell line from BALB/c mouse strain) was provided by Dr. Lyse Norian of the University of Iowa. HMLE (Normal human mammary epithelial cell line), MCF-7 cells (luminal A human breast cancer cell line) and MDA-MB-231 cells (triple-negative human breast cancer cell line) were kindly provided by Dr. Weizhou Zhang of the University of Iowa.

### Animals

Five-week-old C57BL/6NCr and BALB/cAnNCr female mice were used. Mice were sacrificed when they became moribund. Tumor volume (TV) was calculated as: TV = 0.5(length*width^2^) [22]. Tumor latency was calculated as the number of tumor-free injected mice/number of injected mice × 100. To assess morbidity, the following clinical score ranking was used: (0) no abnormal clinical signs, (1) ruffled fur but lively, (2) ruffled fur, activity level slowing, sick, (3) ruffled fur, eyes squeezed shut, hunched, hardly moving, very sick, (4) moribund and (5) dead [23]. Experiments involving mice were approved by the University of Iowa Animal Care and Use Committee (IACUC).

### Mice injections and live animal imaging

Orthotopic mammary tumors were generated by implanting 1.5 × 10^5^ cancer cells in 200 μl of phosphate-buffered saline (PBS) into the mammary fat pad of five-week-old female mice. Prior to imaging, mice were anesthetized, weighed and injected intraperitoneally with D-luciferin. Mice were imaged using the Xenogen IVIS three-dimensional optical imaging system (Caliper Life Sciences, Hopkinton, MA, USA). Luciferase was quantified with Living Image Software (Caliper Life Sciences).

### Histology

Gastrointestinal samples were rolled for processing to allow visualization of mesenteric tumors. Fixed tissues were paraffin embedded, sectioned at 4 μm, and stained with hematoxylin and eosin (H&E). Spleen and lung sections were imaged using a BX51 Olympus microscope (Olympus, Tokyo, Japan). Gastrointestinal slides were scanned with an Aperio ScanScope CS (Aperio Technologies, San Diego, CA, USA).

### Lentiviral transduction

E0771 and 4T1 cells were stably transduced with a construct expressing LV-CMV-firefly luciferase or an empty vector construct using lipofectamine following the manufacturer’s instructions (Life Technologies, Carlsbad, CA, USA). Stable transfectants were then transduced with lentiviral particles carrying BST-2-targeting sh137: CCGGCGCGATCTTGGTGGTCCTGTTCTCGAGAACAGGACCACCAAGATCGCGTTTTTG; sh413: CCGGGCTTGAGAATGAAGTCACGAACTCGAGTTCGTGACTTCATTCTCAAGCTTTTTG; or a non-targeting shControl: CCGGCAACAAGATGAAGAGCACCAACTCGAGTTGGTGCTCTTCATCTTGTTGTTTTT using a previously described protocol [17]. Stable cells were generated by selection with the appropriate drug. The short hairpin RNA (shRNA) constructs were purchased from Sigma-Aldrich (St Louis, MO, USA) (SHCLND-NM_198095) and lentiviral particles were generated at the Gene Transfer Vector Core at the University of Iowa.

### Flow cytometry

Cell monolayers were washed with PBS and treated with Versene (Life Technologies). Single cells were stained with fluorescein isothiocyanate (FITC)-conjugated anti-mouse BST-2 (eBioscience, San Diego, CA, USA), allophycocyanin (APC)-conjugated anti-human BST-2 (BioLegend, San Diego, CA, USA), and appropriate immunoglobulin Gs (IgGs) [16],[17] at 4°C for 1 hour. After washing, cells were incubated with a fluorescent intercalator - 7-aminoactinomycin D (7-AAD) (BioLegend) at 4°C for 30 minutes to assess cell viability. Stained cells were quantified using a FACSCalibur flow cytometer (BD Biosciences, San Jose, CA, USA). At least 10,000 events were collected per sample. Fluorescence-activated cell sorting (FACS) data were analyzed by Flowjo software (TreeStar, Ashland, OR, USA).

### Reverse transcriptase quantitative real-time PCR (RT-qPCR)

Isolation of RNA was accomplished using the RNeasy mini kit (Qiagen, Venlo, Netherlands) according to the manufacturer’s instructions. Equivalent amounts of DNase I (Qiagen)-treated RNA were reverse-transcribed with a high-capacity cDNA reverse transcription kit (Applied Biosystems, Carlsbad, CA, USA). cDNA was amplified with target-specific primers (GAPDH-Forward: 5′-CCCCTTCATTGACCTCAACTACA-3′, Reverse: 5′-CGCTCCTGGAGGATGGTGAT-3′; mouse BST-2-Forward: TCAGGAGTCCCTGGAGAAGA, Reverse: ATGGAGCTGCCAGAGTTCAC; human BST-2 RT^2^ qPCR Primer Assays (SABiosciences, Frederick, MD, USA). RT-qPCR was carried out with an ABI 7500 FAST thermal cycler (Applied Biosystems) as previously described [24].

### Western blot

Western blots were performed as previously described [24]. Blots were probed with anti-BST-2 (Abcam, Cambridge, UK) and anti-GAPDH (Santa Cruz Biotechnology, Dallas, TX, USA) primary antibodies and appropriate IRDye secondary antibodies were used. Band detection and quantification were carried out with the Odyssey Infrared Imaging System (LI-COR Biosciences, Lincoln, NE, USA).

### MEF adhesion assay

Murine embryonic fibroblasts (MEFs) were grown to confluency in 6-well plates. Equivalent numbers (150,000) of cancer cells labeled with PKH67Green fluorescent cell linker, following the manufacturer’s instructions (Sigma-Aldrich), were added to the MEF monolayer and allowed to incubate for 8 hours. Non-adhered cells were washed off and adhered cells imaged. Image J (NIH, Bethesda, MD, USA) was used to quantify the number of PKH67Green-positive cells. In parallel, luciferase-expressing cancer cells were added to MEFs monolayers in 6-well plates for 8 hours. Non-adhered cells were washed off and adhered cells were treated with D-luciferin and imaged using IVIS. For luciferase assay, cancer cells plated in 96-well plate were used for quantitation of luciferase bioluminescence.

### Collagen and fibronectin adhesion assay

Ninety-six-well plates were coated with 50 μg/ml of collagen or fibronectin. Plates were incubated at 37°C for 2 hours. Nonspecific sites were blocked with 40 μl of 2 mg/ml bovine serum albumin (BSA) in PBS. Wells were washed once with PBS. Cancer cells were labeled with PKH67 Green fluorescent cell linker, following the manufacturer’s instructions (Sigma-Aldrich). Labelled cells were added to pre-coated wells (20,000 cells/well) and allowed to adhere for 4 hours. Non-adhered cells were washed off with PBS and plates were read at 485 nm/535 nm (excitation/emission) wavelengths using a Tecan Infinite M200 Pro plate reader (Tecan, Maennedorf, Switzerland). Values are represented as relative fluorescence unit.

### Scratch assay

Confluent monolayers of cancer cells plated in 12-well plates were scratched using a pipette tip. Fresh medium was added to the wells. Cells were allowed to migrate for 0, 6 or 24 hours before fixation (4% paraformaldehyde (PFA) for 45 minutes). Fixed cells were washed (1 × PBS) and imaged with a Nikon Eclipse Ti microscope adjusted with a Nikon digital sight camera (Nikon, Tokyo, Japan). Images were processed and migrated cells counted using Image J software.

### Boyden chamber assay

The apical chamber of 24-well cell culture inserts (Merck Millipore, Billerica, MA, USA) were seeded with previously starved sh137, sh413 or shControl transduced E0771 cells (150,000) in serum-free medium. Culture medium containing 30% FBS was added to the basal chamber of the unit and cells were allowed to migrate through the membranous barrier for 20 hours at 37°C. Non-migrated cells were washed off, migrated cells were fixed with 4% PFA for 5 minutes, washed twice with 1 × PBS, permeabilized with 100% methanol for 25 minutes, labeled with Giemsa stain (for 15 minutes at room temperature) and imaged using a Nikon Eclipse Ti microscope adjusted with an X-cite series 120 LED fluorescence microscope light source and a Nikon digital sight camera. Images were processed using Image J software. Cells from five different fields were counted and averaged.

### Cell invasion assay

The apical chamber of 24-well cell culture inserts (Merck Millipore) were coated with 3 mg/ml of Matrigel (100 μl) (Sigma-Aldrich) and allowed to solidify for 5 hours. A total of 100,000 sh413- or shControl-transduced E0771 or 4T1 cells in serum-free medium were plated on top of the Matrigel layer. Culture medium containing 10% FBS and 5 μg/ml fibronectin (adhesive substrate) (Sigma-Aldrich) was added to the basal chamber of the unit (600 μl) and cells were allowed to invade through the membranous barrier for 24 hours at 37°C. Noninvasive cells were washed off; invasive cells were fixed with 4% PFA, permeabilized with 100% methanol, labeled with Giemsa stain and imaged as described in the previous paragraph. Images were processed using Image J software. Cells from five different fields were counted and averaged.

### MTT assay

A total of 5,000 cells stably expressing sh137, sh413, or shControl were plated in 96-well plates. Cells were then incubated with 5 mg/ml MTT reagent for 3.5 hours followed by addition of MTT solvent (0.1% NP-40 and 4 mM HCl in isopropanol) and rocking for 15 minutes. Absorbance at 590 nm was read using a Tecan Infinite M200 Pro plate reader.

### BrdU assay

A total of 5,000 cells were plated in 96-well plates for 24 hours. Bromodeoxyuridine or 5-bromo-2′-deoxyuridine (BrdU) (Calbiochem, Billerica, MA, USA) assay was carried out according to the manufacturer’s instructions. Absorbance at 450 nm was read using a Tecan Infinite plate reader. In parallel, 150,000 cells were plated in 24-well plates for 24 hours. Cells were incubated with BrdU label (1:2000) for 20 hours, treated with a fixative/denaturing solution (30 minutes) and incubated with an anti-BrdU antibody (1:1000) and rat anti-mouse BST-2 antibody (1:200, eBioscience) for 1 hour at room temperature. Cells were washed and incubated with Alexa Fluor 594 anti-rat (Invitrogen, Waltham, MA, USA) and Alexa Fluor 488 anti-mouse (Invitrogen) secondary antibodies for 30 minutes at room temperature. Cells were stained with 4′,6-diamidino-2-phenylindole (DAPI)-containing Vectashield (Vector Laboratories, Burlingame, CA, USA) and imaged using a Zeiss 710 confocal microscope (Carl Zeiss, Oberkochen, Germany). Images were processed using Image J software. BrdU label, fixative/denaturing solution, and anti-BrdU antibody were from BrdU (Calbiochem) assay.

### Transformation assay

Agar was mixed in Roswell Park Memorial Institute medium (RPMI) with 20% FBS. A total of 500 μl of 0.5% agar was added to 24-well plate and allowed to solidify. Cells were plated at 1,250 cells/well in 500 μl of 0.35% agarose. Some 250 μl of the appropriate growth medium was added on top of the agarose layer. Growth medium was replaced twice a week. Colonies were stained with crystal violet and imaged. Colonies from five different fields were counted and averaged.

### Meta-analysis

Three publically available Gene Expression Omnibus (GEO) datasets GSE4922 [25], GSE21422 [26] and GSE10797 [27] were used to analyze BST-2 expression with respect to tumor size, breast cancer classification and tumor type, respectively. From the GSE4922 dataset, only data from the Affymetrix Human Genome U133A Array were used (Affymetrix, Santa Clara, CA, USA). From these data, only patients from Uppsala (Sweden) who had BST-2 transcript expression from tumor and tumor size data were considered. Patients who had tumor size values higher than 100 mm (one patient, outlier) were excluded. The publicly available GSE21422 dataset was used to determine whether there was a relationship between BST-2 expression and breast cancer classification. BST-2 expression was measured by GeneChip Robust Multiarray Averaging (GC-RMA). All data points were used. The publicly available GSE10797 dataset was used to determine whether BST-2 transcript levels are high in multiple cell types (epithelial and stromal cells) that form the tumor environment. In addition, the publicly available breast-invasive carcinoma (BRCA) data from The Cancer Genome Atlas (TCGA) data portal was used to evaluate the expression of BST-2 and patient survival. The data were downloaded through the University of Iowa’s Institute for Clinical and Translational Science website [28] and through the University of California, Santa Cruz Cancer Browser. Patients who only had BST-2 expression data available from tumor tissues and not normal tissue or vice versa were excluded from the analysis of BST-2 levels in normal vs tumor tissues (100 patients were analyzed). For BST-2 level analysis in different cancer subtypes, primary tumor data was segregated on their different breast cancer subtypes and BST-2 levels were plotted. For survival analysis, primary tumor data were segregated based on BST-2 expression levels. The top 120 (highest BST-2 expressing patients - High) and bottom 120 (lowest BST-2 expressing patients - Low) samples were used for this analysis. A Kaplan-Meier plot (GraphPad Prism 6, GraphPad Software, San Diego, CA, USA) was used to analyze survival of patients expressing different levels of BST-2 in their primary tumor tissues. Median overall survival time and area under the curve (AUC) were calculated using the GraphPad Prism 6 software.

### Statistics

Statistical analysis of significant differences was queried using the GraphPad Prism 6 software. Kaplan-Meier survival plots were analyzed with the Gehan-Breslow-Wilcoxon test using the GraphPad Prism 6 software. A probability (*P*) value of 0.05 or lower was considered significant.

## Results

### BST-2 expression in breast tumor is associated with tumor size, tumor aggressiveness, and host survival

We studied BST-2 expression in different human breast cancer cells compared to normal mammary epithelial cells. Normal mammary epithelial cells did not express high BST-2, however, cancer cell lines exhibited high levels of BST-2 mRNA (Figure S1A in Additional file 1) and protein (Figure S1B in Additional file 1), consistent with a previous report [10], and suggestive of a potential role in mammary oncogenesis.

**Figure 1:**
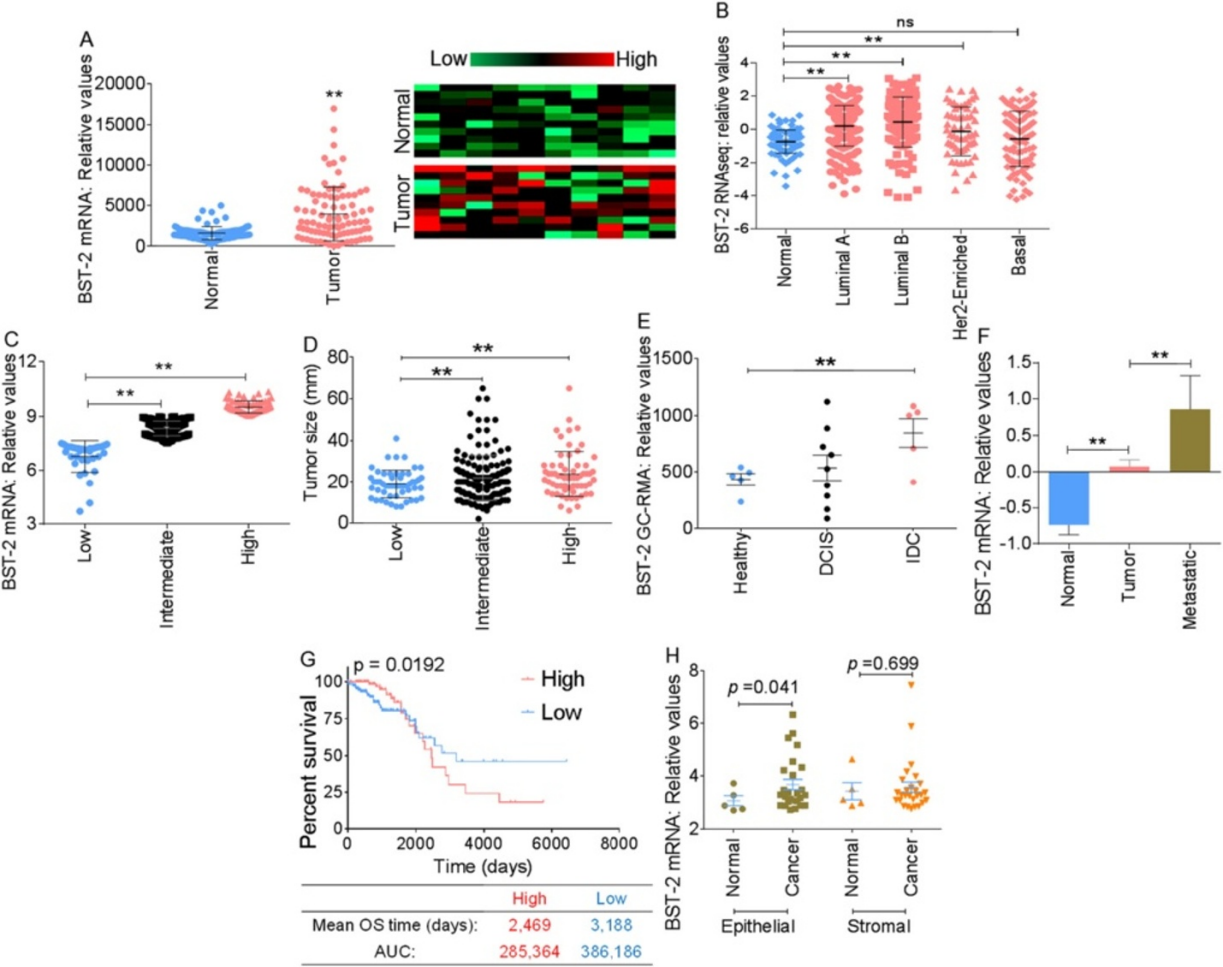
**BST-2 mRNA is prevalent in highly aggressive tumors and associates with patients’ poor survival. (A)** RNA-seq data (n = 100) of paired tumor versus normal breast tissues from The Cancer Genome Atlas (TCGA) breast-invasive carcinoma (BRCA) data portal presented as scatter plot and heat map show that BST-2 is significantly elevated in tumor tissues compared to matched normal breast tissues. **(B)** Levels of BST-2 in tumor tissues of patients bearing different subtypes of invasive breast carcinomas show that BST-2 is upregulated in different breast tumors subtypes with the exception of the basal subtype. **(C)** BST-2 expression in tumors from Uppsala (Sweden) breast cancer patients obtained from GSE4922 was segregated into three BST-2 expression levels (relative units): low = 6.0 to 7.5, intermediate = 7.5 to 9.0, and high = 9.0 to 11.0. **(D)** Tumor size in patients with low, intermediate, or high levels of BST-2 is shown. **(E)** BST-2 GeneChip Robust Multiarray Averaging (GC-RMA) signal scores from healthy, ductal carcinoma *in situ* (DCIS), and invasive ductal carcinoma (IDC) tumor-bearing patients obtained from GSE21422. **(F)** BST-2 levels from normal, primary tumors (tumors), and metastatic tumors (metastatic) of patients bearing invasive breast cancer (TCGA). **(G)** Kaplan-Meier survival analysis using TCGA (BRCA) primary tumor samples segregated into high and low BST-2 levels show a significant link between low BST-2 and patient survival. The median overall survival (OS) time and the area under the curve (AUC) for each group are shown. **(H)** Mammary epithelial and stromal cells obtained from normal and invasive breast cancer patients (GSE10797) show elevated BST-2 expression in cancerous epithelial cells but not in cancerous stromal cells. In all panels, numbers correspond to *P* values. The relative units for BST-2 RNA levels acquired from TCGA and Gene Expression Omnibus (GEO) datasets are SEM-normalized and centralized log2(x + 1). Error bars represent standard deviations and significance was taken at *P* <0.01^**^. ns = not significant.

Meta-analysis of large-scale human breast cancer data from the GEO and TCGA was used to assess the level of BST-2 mRNA in breast tumors. We compared BST-2 expression in paired normal breast tissues versus resected BRCAs from subjects with known clinical outcomes. BST-2 expression was significantly higher in tumor tissues compared to their paired normal breast tissues (Figure 1A). Stratification of TCGA data into different tumor subtypes showed that compared to normal tissues, BST-2 expression was significantly elevated in all tumor subtypes analyzed with the exception of the basal subtype, where the difference did not reach statistical significance (Figure 1B). Of note, the high-grade luminal B tumors expressed more BST-2 mRNA than the low-grade luminal A, human epidermal growth factor receptor 2 (HER2)+, and basal type (Figure 1B).

Large breast tumors have higher BST-2 expression compared to smaller tumors (Figure 1C and D) as revealed by meta-analysis of human breast cancer data using the GEO dataset GSE4922 [25]. Separation of the data into low, intermediate, and high BST-2 levels showed that subjects whose tumors had high BST-2 (Figure 1C) had strikingly larger tumors (Figure 1D) compared to the tumor masses in subjects with intermediate and low BST-2 (Figure 1C and D). These findings were consistent with the premise that high BST-2 levels may be predictive of tumor aggressiveness and reduced patient survival. We therefore investigated this possibility using the GEO dataset GSE21422 [26] containing BST-2 mRNA expression data from normal breast tissues and tumor tissues from ductal carcinoma *in situ* (DCIS) and invasive ductal carcinoma (IDC). As expected, BST-2 expression was higher in the most aggressive form of breast cancer, IDC, compared to DCIS (Figure 1E).

Additionally, analysis of BST-2 expression profile with TCGA dataset segregated into normal, primary tumor, and metastatic tumor revealed that levels of BST-2 in metastatic tumors were highly elevated compared to primary tumors (Figure 1F). Furthermore, Kaplan-Meier model showed that subjects with high tumor BST-2 had significantly reduced survival than those whose tumors had low BST-2 expression (Figure 1G). While patients bearing high BST-2-expressing tumors had median overall survival (OS) time of 2,469 days and AUC of 285,364, subjects with low BST-2-expressing tumors had OS of 3,188 days and AUC of 386,186. These data suggest that BST-2 expression is a strong predictor of survival.

Mammary cancers are epithelial neoplasms and epithelial/stromal interactions are critical in mammary cancer development and progression. To probe into the source of BST-2 in breast tumors, the GEO dataset GSE10797 [27] was used to investigate the pattern of BST-2 expression in epithelial cells versus the surrounding stromal cells. There was no difference in BST-2 levels between stromal cells from tumor and normal mammary tissues (Figure 1H). In contrast, BST-2 expression was significantly higher in epithelial cells from tumor compared to epithelial cells from normal breast tissues (Figure 1H).These data suggest that epithelial cell-intrinsic BST-2 may be a significant contributor of elevated BST-2 in tumor tissues. Together, these results indicate that BST-2 is most prevalent in extremely aggressive tumors and associates with patients’ poor survival.

### Suppression of BST-2 expression in mammary cancer cells prolongs time to primary tumor formation and reduces tumor mass

To establish a system to analyze the functional implication of BST-2 expressed in cancer cells (Figure S2A in Additional file 2), we suppressed BST-2 expression in two murine mammary cancer epithelial cell lines, E0771 cells [29] and 4T1 cells [30]. E0771 cells are syngeneic to C57BL/6 mice while 4T1 cells are syngeneic to BALB/c mice. These models resemble human breast cancer with respect to progression and metastasis [29],[30]. Using BST-2-targeting shRNA (sh137 and/or sh413), we efficiently downregulated BST-2 expression in E0771 and 4T1 cancer cells (Figures S2B to S2E in Additional file 2). A non-targeting shRNA (shControl) was used as control. Both BST-2-targeting shRNA constructs reduced BST-2 expression; but sh413 more efficiently suppressed BST-2. Consequently, sh413-expressing cells were used in all *in vivo* studies.

**Figure 2:**
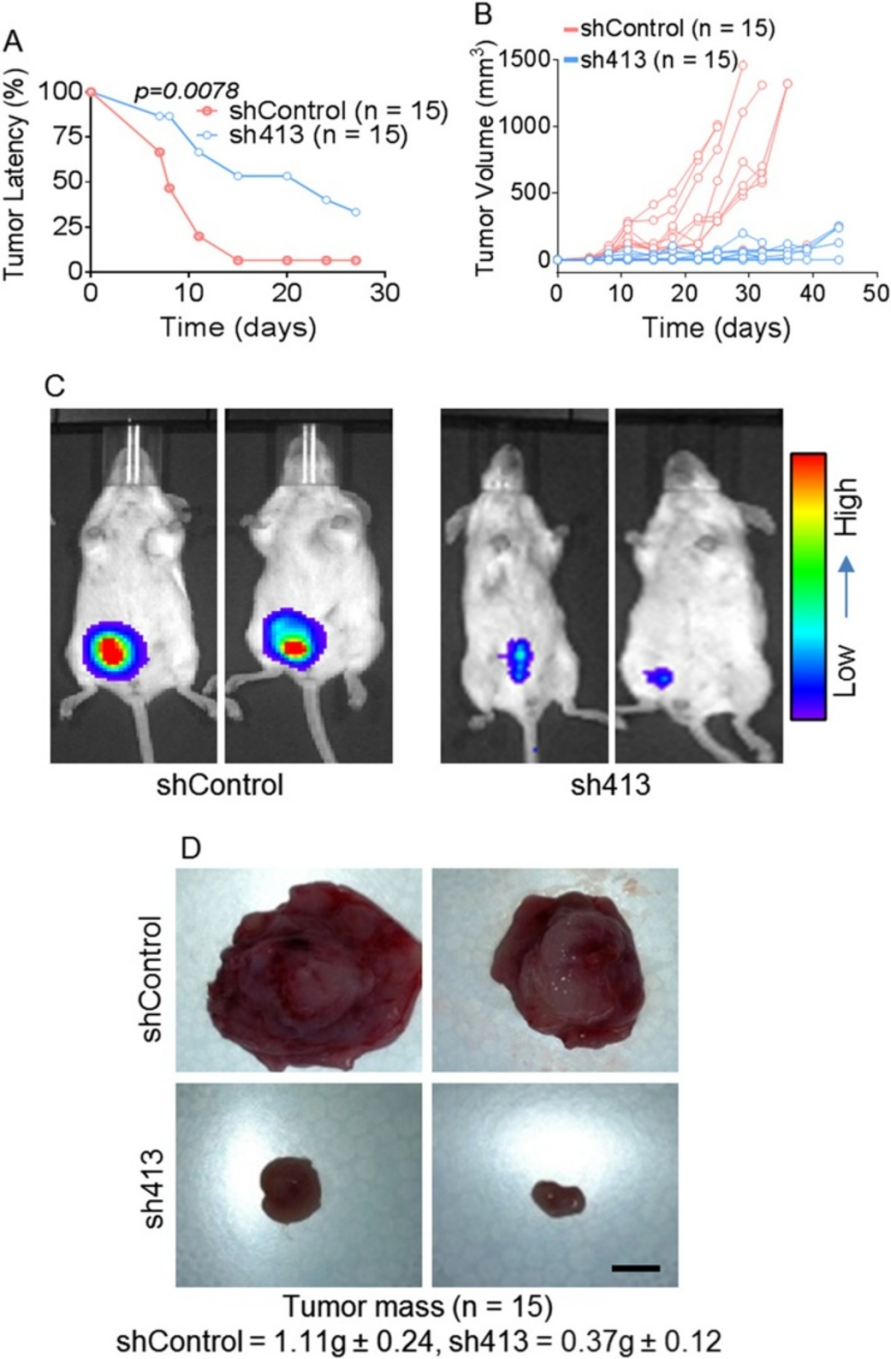
**Suppression of BST-2 in cancer cells increases tumor latency and decreases tumor mass** ***in vivo.*** **(A)** Knockdown of endogenous BST-2 expression in 4T1 cells increases tumor latency computed as (number of tumor-free injected mice/number of injected mice) x100. **(B)** Tumor volume (TV) computed as TV = 0.5 (length*width^2^) over time is significantly reduced when BST-2 is suppressed in 4T1 cells. **(C)** Tumor cells tracked *in vivo* with IVIS imaging system show significant reduction in luciferase expression in BST-2-suppressed sh413 compared to BST-2-expressing shControl injected mice. **(D)** Loss of BST-2 in cancer cells reduced tumor mass. Tumor weight (numbers, g) and gross images obtained at necropsy are shown. All mice implanted with 4T1 shControl or sh413 cells developed mammary tumors with variation in size. Numbers represent average ± SEM. Scale bar = 5 mm.

To determine the effect of BST-2 in primary mammary tumor development, we inoculated BST-2-expressing shControl and BST-2-suppressed sh413 4T1 cells into the mammary fat pads of BALB/c mice and evaluated tumor growth. 4T1 cells formed primary tumors in the mammary fat pad prior to metastasis [30]. We observed increased mammary tumor latency (Figure 2A) and delayed mammary tumor onset (Figure 2B) in mice implanted with BST-2-suppressed sh413 cells compared to shControl cells. Tumor volume over time was significantly lower in sh413 tumors compared to shControl tumors (Figure 2B). Because 4T1 cells were tagged with luciferase, we tracked cancer cells *in vivo* by IVIS imaging. As expected, luciferase intensity (photons/sec) was much lower in mice implanted with sh413 cells compared to shControl-implanted mice at the site of injection (Figure 2C). Inoculation of mice (n = 15) with BST-2-expressing shControl cells resulted in massive mammary tumors with an average tumor mass of 1.11 g ± 0.24 (Figure 2D). This result was in stark contrast to mice (n = 15) inoculated with BST-2-suppressed sh413 cells that developed significantly smaller tumors averaging 0.37 g ± 0.12 in weight (Figure 2D).

The effect of BST-2 in tumor development was also evident in the E0771-C57BL/6 model (Figure S3 in Additional file 3). E0771 cells are highly metastatic [29]. Expression of BST-2 in E0771 cells had a tumor-enhancing effect similar to the one observed with the 4T1 cells. BST-2-expressing E0771 cells (shControl) showed significant decrease in tumor latency compared to BST-2-suppressed E0771 cells (sh413) (Figure S3A in Additional file 3). Together, these data suggest that downregulation of BST-2 expression in breast cancer cells delays mammary tumor onset and may impair the ability of primary tumors to thrive.

### Knockdown of BST-2 in cancer cells decreases metastases to the lung and other distal sites

**Figure 3:**
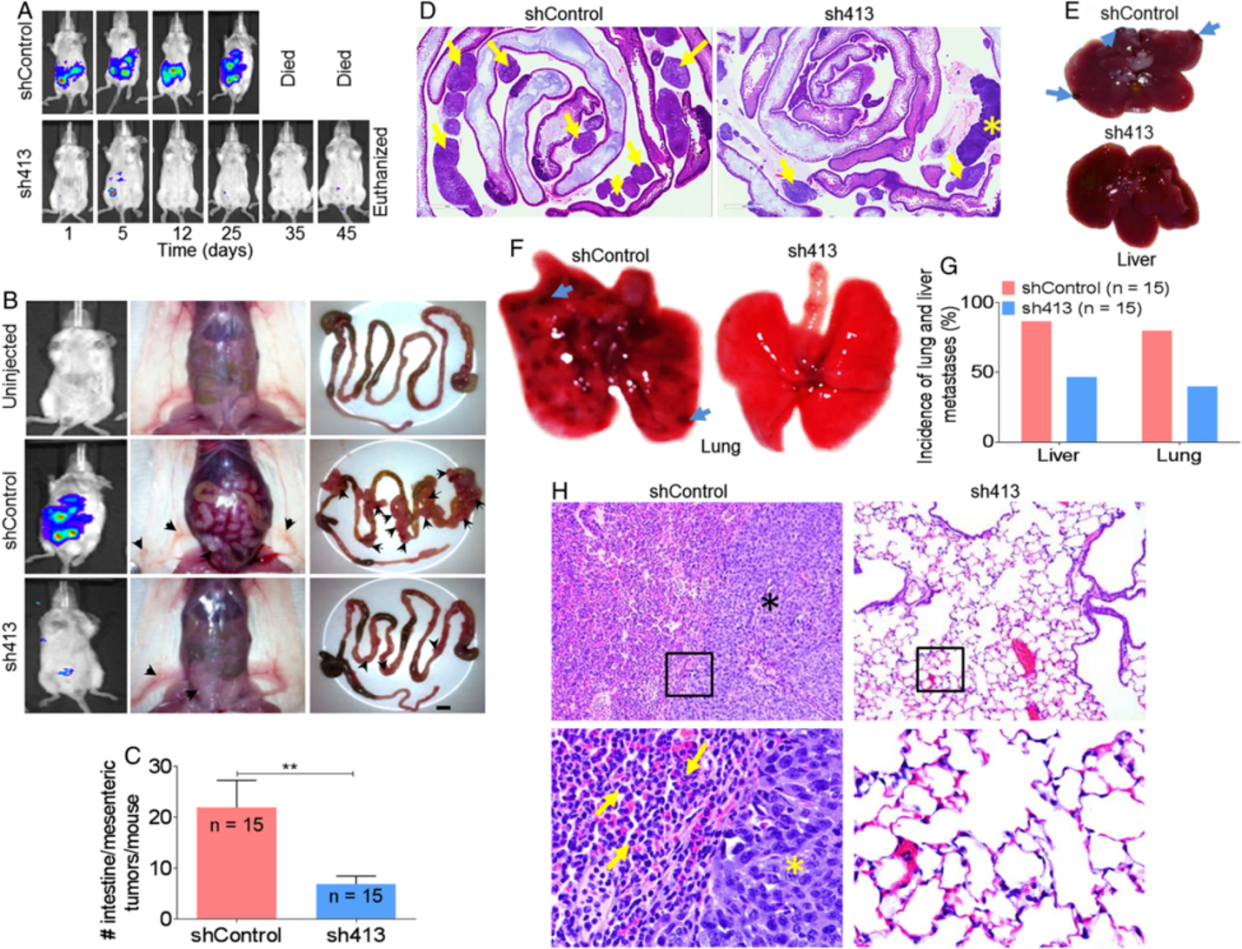
**Down-modulation of BST-2 in cancer cells reduces mammary cancer metastases. (A)** Representative images of tumor cells tracked *in vivo* with IVIS imaging at different time points. Images show higher luciferase bioluminescence in 4T1 shControl-injected mice compared to sh413-injected mice. **(B)** Representative luciferase bioluminescence accompanied with abdominal and gastrointestinal tract (GI tract) gross images of uninjected (upper panel), shControl-implanted (middle panel), and sh413-implanted mice (lower panel). Arrow heads point to mammary tumors (middle column) and intestinal/mesenteric tumors (right column). Scale bar = 5 mm. **(C)** Number of secondary tumors in intestine/mesentery plotted as average of all mice. **(D)** Representative intestine/mesentery histology images from 4T1 shControl and sh413-injected mice confirming increased mesenteric tumors (arrows) in shControl mice compared to sh413-injected mice. A mesenteric lymph node is demarcated by an asterisk (not to be confused with a tumor mass). **(E)** Representative gross liver images of 4T1 shControl and sh413-injected mice. Arrows are pointing to tumors. **(F)** Representative gross images of lungs showing visible pulmonary nodules (arrows) in shControl-implanted mice. **(G)** Percent incidence of liver and lung metastases. **(H)** Lung histology from shControl (upper left) and sh413 (upper right) injected mice. Lung from the 4T1 shControl mice had multiple large tumors (tumors demonstrated by asterisk) and marked infiltration of the alveolar septa and alveolar spaces by neutrophils (yellow arrows). Boxed regions are shown at higher magnification (40X) for shControl (lower left) and sh413 (lower right). Error bars represent standard deviations and significance was taken at *P* <0.01^**^.

E0771 and 4T1 cells metastasize to liver, bone, lung, and intestine [29],[31]. Thus, we investigated whether BST-2 enhances the metastatic potential of primary tumor cells. As expected, all mice implanted with BST-2-expressing shControl 4T1*luc* cells showed early onset and progressive increase in bioluminescence. The increase in bioluminescence signal intensity over time suggests progression and metastasis of cancer (Figure 3A, upper panel, Figure 3B, left, middle panel). Indeed, BST-2-expressing shControl cells formed primary tumors quickly and developed metastatic lesions that could be detected by bioluminescence imaging [32]. In striking contrast, BST-2-suppressed 4T1 cells (sh413) exhibited delayed onset of luciferase bioluminescence and disappearance of expression as measured over 45 days (Figure 3A, lower panel; Figure 3B, left, bottom panel). Unlike shControl-implanted mice that developed severe abdominal hemorrhage and intestinal/mesenteric tumors (Figure 3B, center, middle and right panels), sh413-implanted mice did not develop hemorrhage and had few intestinal/mesenteric tumors (compare Figure 3B, uninjected - upper panel with Figure 3B, sh413-injected - lower panel). Metastasis to the intestine and mesentery were significantly reduced from about 21 tumors in shControl mice (Figure 3B, middle right panel and Figure 3C) to six tumors in sh413 mice (Figure 3B, bottom right panel and Figure 3C). Histology confirmed increased intestinal/mesentery tumors in shControl-implanted mice compared to sh413-implanted mice (Figure 3D, arrows). These findings were confirmed with the highly metastatic E0771 cells. Mice (n = 10) implanted with BST-2-expressing E0771 cells (shControl) had higher bioluminescence and increased intestinal/mesenteric tumors compared to mice implanted with BST-2-suppressed sh413 cells (Figures S3B to S3D in Additional file 3).

Importantly, gross images showed that compared to sh413 cells, shControl cells resulted in significant metastases to the liver (Figure 3E) and lung (Figure 3F). Lungs from shControl-implanted mice were laden with pulmonary nodules, suggesting pulmonary metastases (Figure 3F, arrows). The incidence of metastases to the liver was 86% and 46%, and 80% and 40% to the lung for 4T1 shControl and sh413 cells respectively (Figure 3G). Similar trends in liver and lung metastases were observed with E0771 cells (Figure S3E in Additional file 3). Furthermore, histologic analyses confirmed large metastatic nodules in the lung of mice implanted with 4T1 shControl cells (Figure 3H, asterisk). Additionally, there were clustered and scattered tumor cells throughout the lung interstitium with marked infiltration of neutrophils within the alveolar septa and alveolar spaces of lungs from shControl-injected mice (Figure 3H, arrows).

To test whether the reduced metastasis observed in mice bearing tumors from BST-2-suppressed cells reflect a delay in metastasis due to delayed primary tumor growth and differences in tumor size, we performed a linear regression analysis for correlation between primary tumors and metastatic growth. However, we found no correlation between primary tumor and lung or primary tumor and intestinal/mesentery metastases in our mouse models (not shown). These results show that BST-2 expression promotes mammary tumor metastasis to distal sites.

### BST-2 expression in mammary cancer cells is associated with poor clinical outcome and significant morbidity in tumor-bearing mice

**Figure 4:**
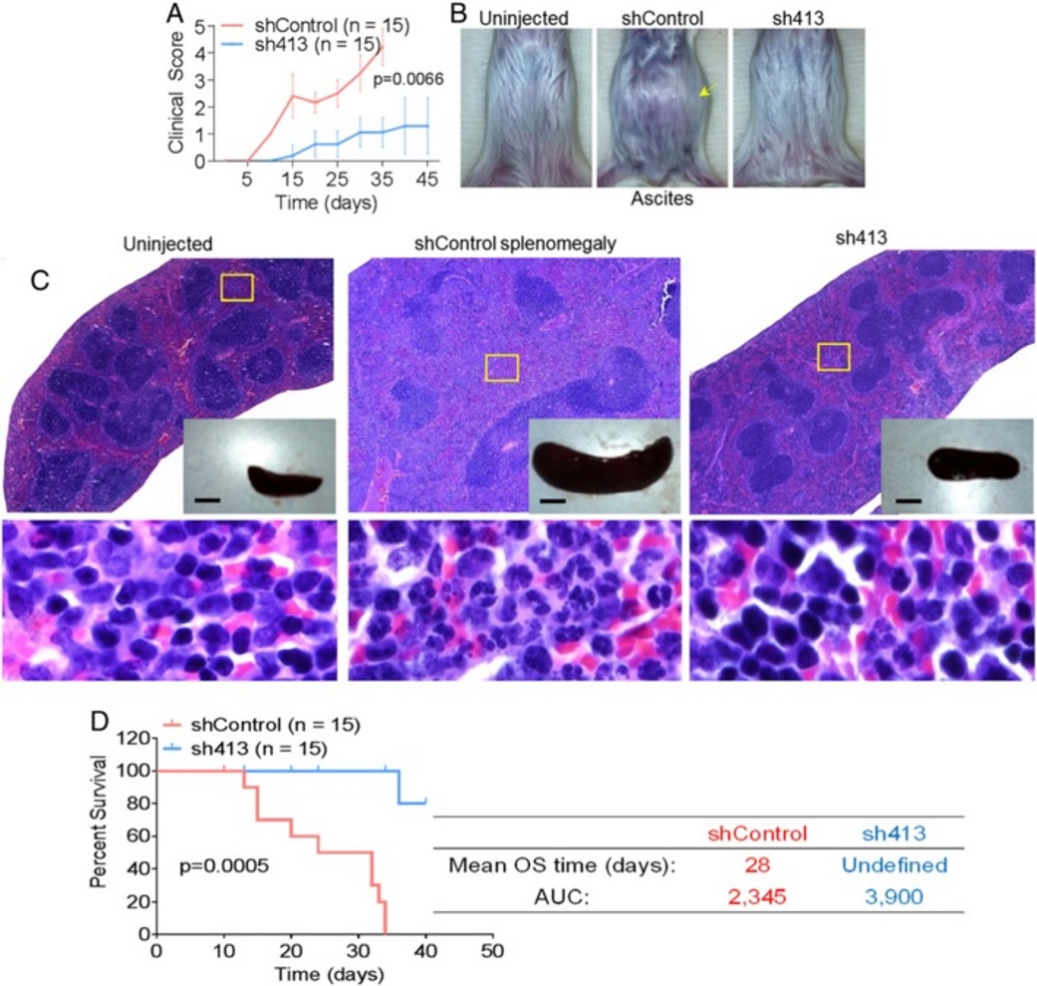
**BST-2 expression in cancer cells is a strong prognostic factor for morbidity and overall survival. (A)** Clinical score plot of mice implanted with 4T1 BST-2-expressing shControl and BST-2-suppressed sh413 cells. Clinical signs were scored as follows: 0 = no abnormal clinical signs; 1 = ruffled fur but lively; 2 = ruffled fur, activity level slowing, sick; 3 = ruffled fur, eyes squeezed shut, hunched, hardly moving, very sick; 4 = moribund; 5 = dead [23]. **(B)** Representative gross images of the abdomen of uninjected (left), shControl-implanted (middle), and sh413-implanted (right) mice. Arrow points to metastatic ascites (middle panel). **(C)** Representative splenic gross images (top panel insets) and spleen histology at low magnification (4X, top panel). Boxed regions are shown at higher magnification (60X) from uninjected (left panel), shControl (middle panel), and sh413 (right panel) injected BALB/c mice (bottom panels). There was marked expansion of red pulp due to granulocytic hyperplasia in the shControl spleen with slightly increased number of granulocytes in the red pulp of the sh413 spleen. Scale bar = 5 mm. **(D)** Kaplan-Meier survival plot of mice implanted with BST-2-expressing shControl and BST-2-suppressed sh413 4T1 cells. Numbers are *P* values and error bars represent standard deviations. Median overall survival (OS) time and the area under the curve (AUC) are shown for each group.

Pronounced effect on morbidity was observed in mice bearing shControl-induced tumors compared to their counterparts bearing sh413-induced tumors. Specifically, mice implanted with BST-2-expressing 4T1 cells developed hypothermia more rapidly and to a higher extent than mice implanted with BST-2-suppressed sh413. Ruffled hair, shallow breathing, and prostration were observed in shControl-implanted mice but not in sh413-implanted mice (Figure 4A). Furthermore, mice implanted with BST-2-expressing shControl 4T1 cells developed malignant ascites (Figure 4B, middle panel) and severe splenomegaly (Figure 4C, middle panel, inset). Remarkably, 14 out of 15 mice implanted with BST-2-suppressed 4T1 cells (sh413) were spared of ascites (Figure 4B, compare left and right panels) and splenomegaly (Figure 4C, compare left and right panel insets). Grossly, spleens from shControl mice were markedly enlarged (Figure 4C, inset). Histologically, the splenic red pulp of shControl-implanted mice was markedly expanded by increased immature and mature granulocytes indicative of increased granulopoiesis (Figure 4C, shControl, lower panel). In contrast, there was a slight increase in red pulp granulocytes in the spleen of sh413-bearing mice (Figure 4C, sh413, lower panel).

Similar to the 4T1 model, clinical manifestations of disease were delayed in BST-2-suppressed sh413 E0771-bearing mice (Figure S4A in Additional file 4). Suppression of BST-2 in E0771 cells prevented the development of malignant ascites in all (n = 10) sh413 bearing mice compared to shControl-bearing mice (Figure S4B, upper panel in Additional file 4). Moreover, BST-2-suppressed E0771 (sh413)-bearing mice did not develop shock (assessed by the appearance of pale digits on forelimbs) as was observed in all E0771 shControl-bearing mice (Figure S4B, lower panel in Additional file 4) and as previously shown in the E0771 model [29]. These results show that expression of BST-2 in cancer cells accelerates disease progression in tumor-bearing mice.

### BST-2-expression in cancer cells results in poorer survival of tumor-bearing mice

Because human breast cancer patients bearing tumors with high BST-2 mRNA have lower survival, we directly evaluated the role of BST-2 expression in cancer cells on the survival of tumor-bearing mice. Kaplan-Meier survival curve analysis reveals that mice implanted with BST-2-suppressed sh413 4T1 or E0771 cells have a statistically significant prolongation in survival compared with BST-2-expressing shControl-implanted mice (Figure 4D (4T1) and Figure S4C (E0771) in Additional file 4). Improvement in survival was more pronounced in the 4T1 model because all (n = 15) mice implanted with 4T1 shControl cells died on average 25 days post implantation. Surprisingly, 14 out of 15 mice implanted with 4T1 sh413 cells survived and were euthanized at the end of the experiment (day 45). One out of 15 4T1 sh413-implanted mice was sacrificed on day 37 post implantation due to tumor-associated morbidity (Figure 4D). The OS and AUC for 4T1 shControl-bearing mice were 28 days and 2,345 compared to sh413-bearing mice with undefined OS and 3,900 AUC. Additionally, mice implanted with E0771 shControl cells died at approximately 16 days post implantation compared to their E0771 sh413 cells-implanted counterparts that averaged 23 days post implantation (Figure S4C in Additional file 4). The OS and AUC of E0771 shControl mice were 16 days and 1,965 respectively, while E0771 sh413-implanted mice have 23 days OS and 2,679 AUC. Together with the human survival data presented in Figure 1G, our results support the premise that BST-2 expression in mammary cancer cells may be a predictor of host survival.

### Intrinsic BST-2 in mammary cancer epithelial cells modulates cancer cells adhesion

**Figure 5:**
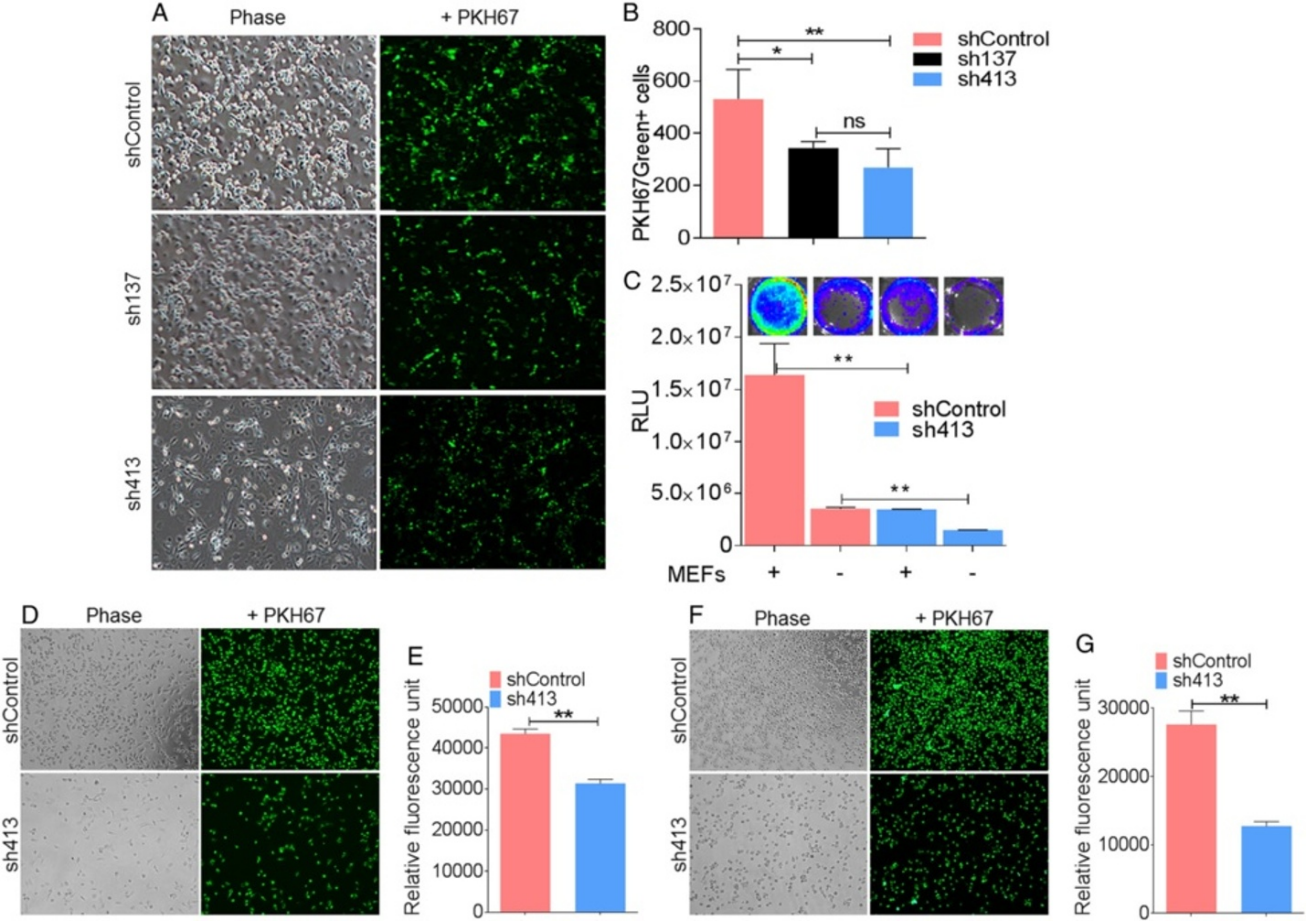
**Elevated mammary tumor cell BST-2 regulates cancer cell adhesion.** PKH67Green-labeled BST-2-expressing shControl and BST-2-suppressed sh137 and sh413 E0771 cells were allowed to adhere to murine embryonic fibroblasts (MEFs). **(A)** Representative confocal images of adhered cells and **(B)** Image J quantification of PKH67Green + cells per field (n = 5) are shown. Images were taken at 20X. **(C)** Analysis of cancer cell adhesion to MEF by quantification of luciferase bioluminescence shControl and sh413 cells. Luciferase assay quantified expression represented as relative light units (RLU) and images (inset) were taken with the IVIS three-dimensional optical imaging system and analyzed with Living Image Software. **(D)** Representative images and **(E)** quantification of shControl and sh413 4T1 cells adhered to collagen-coated plates. **(F)** Representative images and **(G)** quantification of shControl and sh413 4T1 cells adhered to fibronectin-coated plates. Images were taken at 10X. Experiments were performed multiple times with similar results. Error bars represent standard deviations and significance was taken at *P* <0.05^*^, <0.01^**^. ns = not significant.

The striking effects of BST-2 on tumor growth and metastasis led us to define the cellular basis for BST-2 effect on breast tumorigenesis. One characteristic feature of cancer cells is their ability to adhere to and recruit other cells, such as cancer-associated fibroblasts (CAFs) to promote formation of primary tumors [33]. To determine the role of BST-2 in cancer cell adhesion, E0771 cells with varying BST-2 levels were labeled with the fluorescent cell linker PKH67Green dye and added onto confluent monolayers of MEF. We found that cancer cell BST-2 facilitated cancer cell adhesion to fibroblasts as revealed by confocal microscopy (Figure 5A) and Image J quantification (Figure 5B) of PKH67Green + cells adhered to MEFs. Large numbers of the BST-2-expressing (shControl) adhered to fibroblasts compared to cells with intermediate BST-2 (sh137) and low BST-2 (sh413) respectively. In parallel, a similar experiment was performed with luciferase expressing shControl or sh413 cancer cells added onto confluent MEFs. Consistent with the PKH67Green result, suppression of BST-2 decreased the ability of cancer cells to adhere to supporting cells. Compared to shControl cells, lower luciferase bioluminescence was observed in wells containing sh413 both in a luciferase assay (Figure 5C) and by IVIS 200 imaging of luciferase bioluminescence (Figure 5C, inset). To confirm the adhesion result, we analyzed the effects of BST-2 knockdown on adhesion to the extracellular matrix (ECM) supportive substrates collagen and fibronectin [34] using pre-coated plates. As expected, BST-2-suppressed sh413-expressing 4T1 cells had reduced adhesive capability to collagen (Figure 5D and E) and fibronectin (Figure 5F and G) compared to BST-2-expressing shControl cells. These results indicate that BST-2 regulates adhesion of breast cancer cells to CAFs and to ECM proteins.

### BST-2 depletion reduces anchorage-independent growth

**Figure 6:**
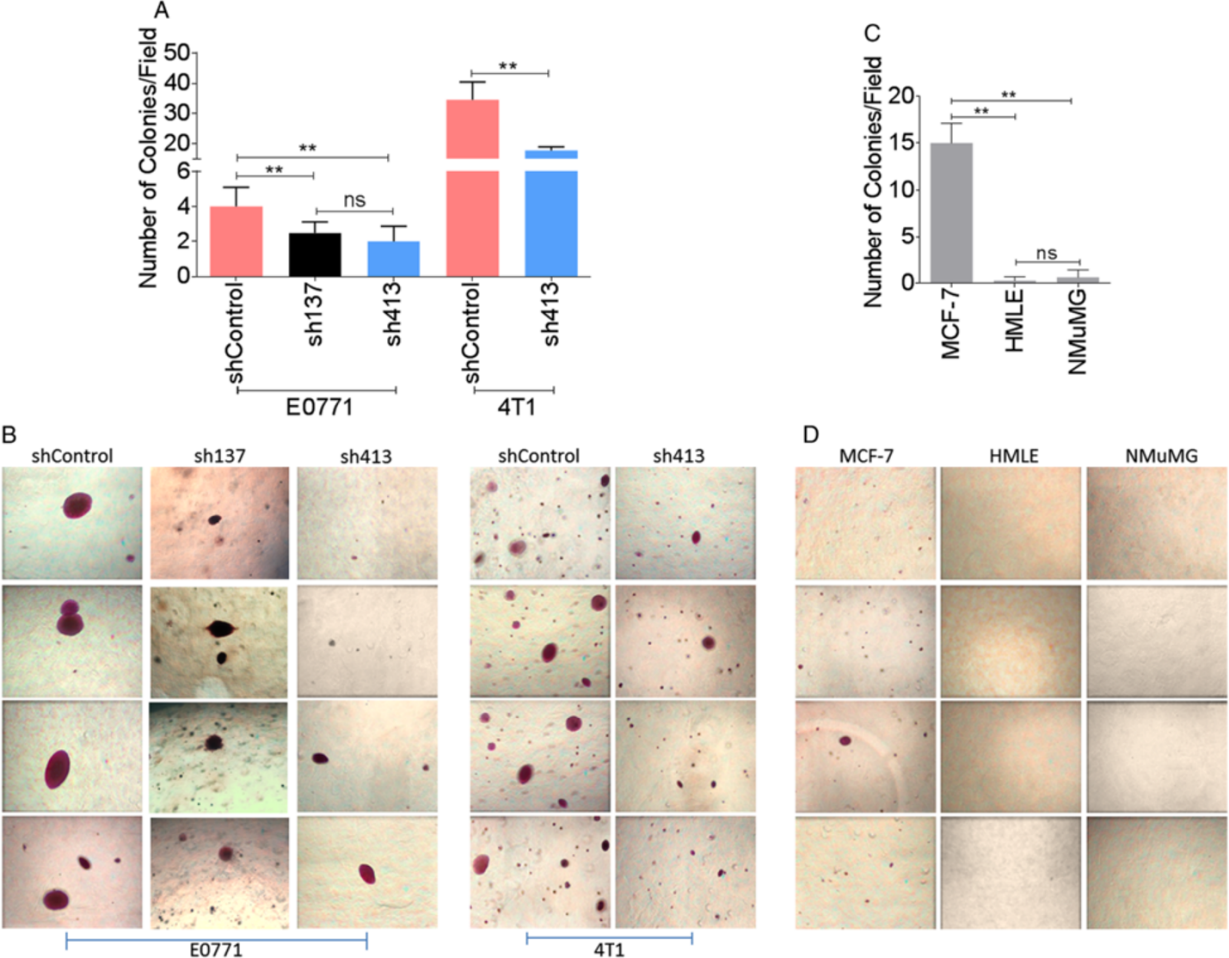
**Suppression of BST-2 expression in cancer cells results in anchorage-independency. (A and C)** Quantification of the number of colonies formed in soft agar by BST-2-expressing shControl, BST-2-suppressed sh137 and sh413 murine cells, MCF-7 (positive control), HMLE and NMuMG cells following a 20-day transformation assay. The bar represents number of colonies per field (n = 5) for each cell type. Error bars correspond to standard deviations. Significance was taken at *P* <0.001^**^. **(B and D)** Representative images of crystal violet-stained colonies from a soft agar assay showing anchorage-independent growth of cancer cells. Clones were imaged at 4X. Experiments were repeated multiple times with similar results. ns = not significant.

Adaptation to new environment is a hallmark of aggressive tumors. To survive, cancer cells are able to grow and expand in the absence of attachments by overcoming anoikis [35]. Because BST-2-expressing shControl cells metastasized more efficiently than BST-2-suppressed sh413 cells *in vivo* (Figure 3), we used a soft agar colony formation assay to examine the possibility that BST-2 is important for anchorage-independent growth of mammary cancer cells. As expected, we observed reduced colony numbers (Figure 6A) and colony size (Figure 6B) in mammary cancer cells with suppressed BST-2 (intermediate-sh137 and low-sh413) compared to cells expressing high BST-2 (shControl). MCF-7 cells, known to form colonies [36],[37], were used as positive control (Figure 6C and D) while normal human (HMLE) and murine (NMuMG) mammary epithelial cells were used as negative controls (Figure 6C and D). Interestingly, overexpression of BST-2 in low BST-2-expressing MCF-7 cells (Figure S5A in Additional file 5) increased MCF-7 colony size relative to empty vector control (Figure S5B and S5C in Additional file 5). These data suggest that suppression of BST-2 expression may diminish *in vivo* tumorigenicity of otherwise highly tumorigenic cancer cells by reducing anchorage-independence of tumor cells, thus preventing expansion of tumor cells, invasion to adjacent tissues, and dissemination throughout the body.

### BST-2 expression promotes cancer cell migration and invasion

**Figure 7:**
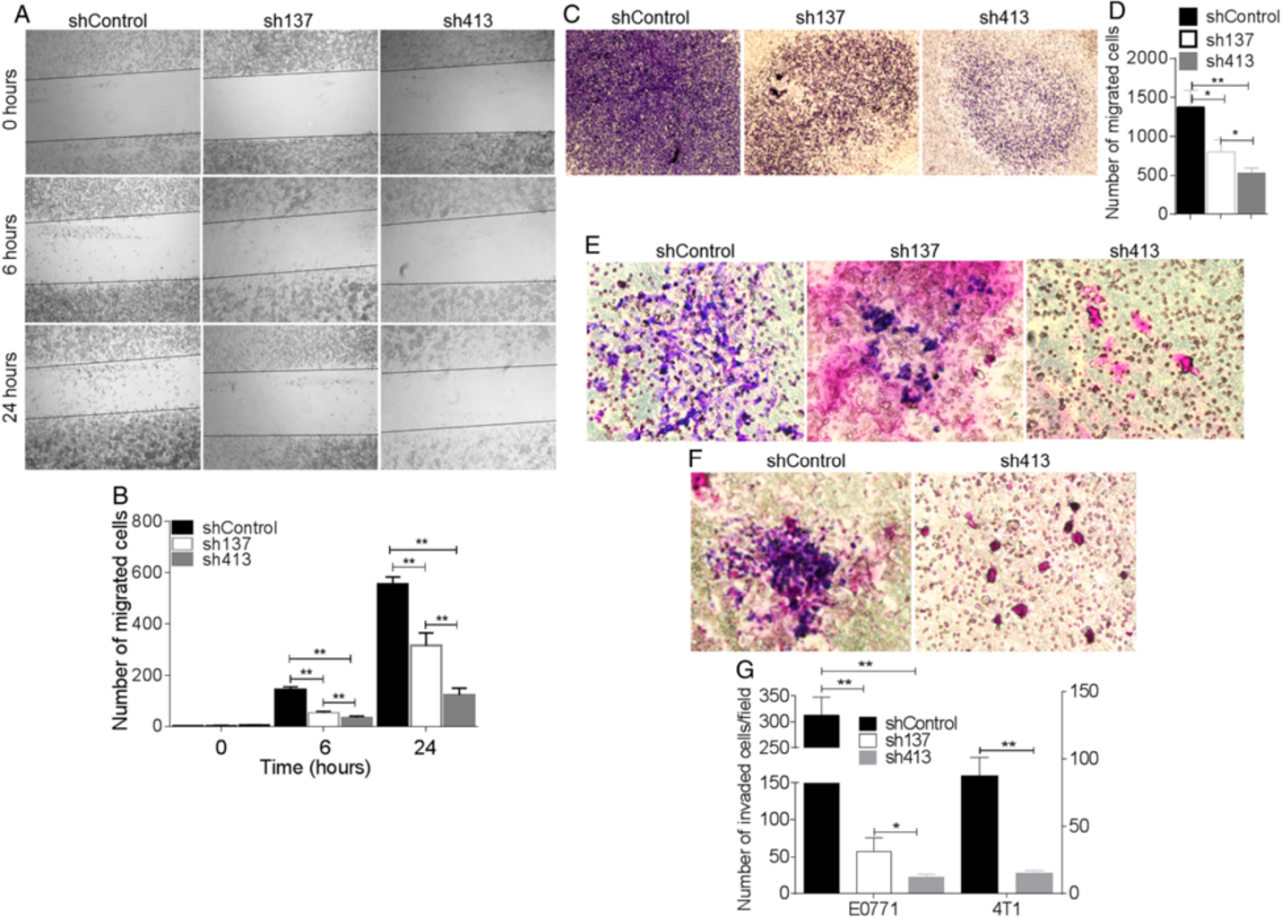
**Endogenous BST-2 expressed in mammary cancer cells controls cell migration and invasion. (A)** Representative images of cell migration performed by scratch assay. Suppression of BST-2 expression by sh137 and sh413 reduced rate of cancer cell migration into the scratch wound. **(B)** Quantification of cells that migrated into the scratch wound at 0, 6, and 24 h. **(C)** Migration assay by Boyden chamber. Representative images taken at 4X. **(D)** Migrated cells were imaged and the number of migrated cells counted with Image J. Similar results were obtained with 4T1 shControl and sh413 cells. **(E)** BST-2-expressing shControl and BST-2 suppressed sh137 and sh413 E0771 cells and **(F)** shControl and sh413 4T1 cells were plated in low-serum medium on Matrigel-coated cell inserts and allowed to migrate for 24 h. Cells were stained with Giemsa stain. Representative images taken at 20X are shown. **(G)** Quantification of cells that invaded through Matrigel. Cells from five different fields were counted and averaged. Error bars corresponds to standard deviations. Significance was taken at *P* <0.001^**^ and *P* <0.05^*^. Experiments were repeated multiple times with similar results.

Following the formation of primary tumors, a subpopulation of cancer cells acquires a metastatic phenotype that allows them to migrate to distant tissues [38]. Our *in vivo* study revealed that expression of BST-2 may promote tumor growth at secondary sites (Figure 3). Because cancer cell migration and invasion are key to metastasis, including dissemination of tumor cell into the lymphatic and blood vessels, and subsequent extravasation of tumor cells into secondary organs [39],[40], we evaluated the effect of BST-2 on cancer cells migration using classical migration scratch assay with the cell comb scratch assay. Cancer cells suppressed of BST-2 (sh137 and sh413) lost their ability to migrate to the scratched wounds compared with those expressing BST-2 (shControl) at both 6 h and 24 h time points (Figure 7A and B). The rate of cell migration into the wound opening was reduced in line with level of BST-2 expression, thus, sh137 cells showed some migration (Figure 7A, column 2, Figure 7B) while sh413 cells lost the ability to migrate (Figure 7A, column 3, Figure 7B). The rate of migration was quantified by blind-counting of at least five different fields. In parallel, we performed migration assay on MCF-7 cells overexpressing BST-2. MCF-7 cells overexpressing BST-2 had higher migratory rate compared to vector control cells (Figure S5D in Additional file 5). These results show that the rate of cancer cell migration is strongly associated with BST-2 levels.

To further evaluate the role of BST-2 in cancer cell migration, we employed commercially available Boyden chamber assays. Equal numbers of shControl, sh137, and sh413-expressing cells were plated in the apical chamber of cell culture inserts. Serum-containing medium was added to the basolateral chamber. Compared to cells with high BST-2 expression (shControl), suppression of BST-2 with sh137 and sh413 significantly reduced rate of cell migration (Figure 7C and D).

Although BST-2 increased rate of cell migration into the scratch wound, we found no BST-2-dependent difference in rate of wound closure at 6 h and 24 h time points, suggesting that cells with high (shControl) and suppressed BST-2 (intermediate-sh137 and low-sh413) expression may proliferate equally. Indeed, proliferation assay examining rate of BrdU incorporation into cells showed that endogenous BST-2 had no effect on cell proliferation (Figure S6A and S6B in Additional file 6). This result is in contrast with a previous study that showed that exogenous overexpression of BST-2 promotes cell proliferation [21]. It is likely that the differences in results are due to different experimental systems or cells. In our study, the lack of BST-2 effect on cell proliferation, upon BST-2 knockdown, may not be due to cell viability because metabolic activity of MTT revealed that both BST-2-expressing and BST-2-suppressed E0771 cells were equally viable (Figure S6C in Additional file 6). However, in 4T1 cells, BST-2 knockdown increased cell viability (Figure S6D in Additional file 6). These data suggest that the effects of BST-2 in colony formation and migration (Figures 6 and 7, respectively) cannot be explained by differences in cell viability or cell proliferation *in vitro*. Although we did not observe BST-2-endowed growth advantage in our two-dimensional culture, we cannot rule out the possibility that BST-2 may promote cell proliferation in soft agar or *in vivo*.

In order to metastasize, cancer cells have to migrate and invade the basement membrane. Hence we investigated the ability of BST-2 to promote cancer cell invasion using a Matrigel model. BST-2-expressing (shControl) and BST-2-suppressed (intermediate-sh137 and low-sh413) cells were allowed to invade into the Matrigel for 24 h. As shown in Figure 7E, F, and G, significantly higher numbers of BST-2-expressing shControl cells invaded into the Matrigel compared to BST-2-suppressed sh137 and sh413 cells in that order. An enhancement in cancer cell invasion also resulted when BST-2 was overexpressed in MCF-7 cells (Figure S5E in Additional file 5). These experiments demonstrate that BST-2 expression is crucial for cancer cell invasion and that suppressing BST-2 expression in cancer cells reduced the ability of the cells to invade the basement membrane.

## Discussion

Host innate immune response is critical for surveillance against pathogens and tumors. However, genes involved in immune response may serve as a double-edged sword in pathogenesis and tumorigenesis. As an innate immunity antiviral gene, BST-2 positively regulates NF-кB activation [14],[15] and its expression is induced by types I and II interferons [16]. Increased expression of BST-2 retains budding viruses to the cell plasma membrane [15],[16] and inhibits virus replication [17],[41]. However, elevated levels of BST-2 in cancer cells have pro-tumor functions [10],[20]. In this study, we demonstrated that BST-2 expressed in cancer cells promoted breast cancer development and progression by altering the behavior of cancer cells. Meta-analysis of TCGA (BRCA) human data that showed that BST-2 is most significantly associated with luminal B tumors, invasive ductal carcinoma, and metastatic tumors imply that BST-2 in cancer cells could be a prognostic factor for highly aggressive cancers. It is known that luminal B tumors are associated with larger tumor mass [42] and patients bearing this tumor subtype have significantly worse disease-free survival compared to patients with luminal A tumors [43]. In our meta-analysis study, we found that human breast tumors with elevated BST-2 mRNA are larger, more aggressive, and patients bearing such tumors have poorer survival. This association study was validated in our mouse model experiments.

Mouse models have contributed to understanding breast oncogenesis [30]. In our studies, we used two syngeneic mouse models to allow investigation of the contribution of cancer cell BST-2 in mammary tumorigenesis in different backgrounds in the context of an intact immune system. Implantation of BST-2-expressing 4T1 or E0771 cells into syngeneic BALB/c or C57BL/6 mice respectively revealed that BST-2 in cancer cells is disease modifying. However, suppressing BST-2 expression decreased the onset of primary mammary tumor growth thereby increasing tumor latency, and decreasing tumor cell metastases and growth at distal sites, as in lung colonization.

Whether the decrease in metastasis observed in mice bearing tumors from BST-2 suppressed cells is a direct result of reduced tumor size or delayed metastasis is unknown. However, our *in vitro* studies that showed that cells with suppressed BST-2 have reduced adhesion, anchorage-independent growth, migration, and invasion supports a role for BST-2 in promoting tumor growth at distal sites, because these cancer cell behaviors are critical for metastasis [39],[40]. In addition, the lack of correlation between formation of primary tumor and lung or intestinal/mesentery colonization in our mouse model suggests that BST-2 may differentially promote tumor growth at the primary and secondary sites.

In addition to increased tumor growth at the primary and secondary sites, mice bearing BST-2-expressing 4T1 shControl cells developed malignant ascites and splenomegaly or ascites and shock in the case of E0771 shControl cells-bearing mice. Ascites in tumor-bearing mice may result from the accumulation of fluid in the peritoneal cavity due to the spread of cancer cells [44]. Ascites is associated with increased vessel permeability and decreased lymphatic drainage [45]. Indeed, human patients with cancer-associated ascites have poor prognosis [46]. It is intriguing that BST-2-suppressed sh413-injected mice had a delayed occurrence (E0771 cells) or absence (4T1 cells) of ascites.

Aside from ascites, mice implanted with 4T1 BST-2-expressing cells but not BST-2-suppressed cells developed severe splenomegaly with expanded splenic red pulp, suggestive of increased granulopoiesis. In mice, the spleen is a normal site of hematopoiesis and reactive hematopoiesis. Thus, the splenic granulocytic hyperplasia in shControl mice is the result of reactive hematopoiesis secondary to granulocyte recruitment to the site of tumor. In humans, splenomegaly can be the result of extramedullary hematopoiesis (the spleen is not a normal site of hematopoiesis in humans) but more commonly a result of cancer cell metastases due to hematogenous disease [47]. Malignant ascites and splenomegaly are manifestations of end-stage events in many cancers including breast cancers [48],[49] and is linked to poor prognosis in tumor-bearing hosts.

BST-2 expression in tumor tissues is positively associated with hosts’ survival. In mice, tumors induced by BST-2-expressing shControl cells were associated with poor survival. The observed difference in survival between 4T1 cells and E0771 cells models could be attributed to (i) the level of aggressiveness of the cells; with E0771 cells being more metastatic [29] than 4T1 cells [30], and (ii) the level of BST-2 in the different cancer cells. We found that, BST-2 expression increased with tumor aggressiveness in human patients. BST-2 expression was highest in the highly aggressive IDC compared to DCIS tumors. Moreover, metastatic tumors expressed more BST-2 than primary tumors.

Although the source of elevated BST-2 in breast tumors is unknown, our data suggest that BST-2 expression in breast epithelial cells derived from breast tumors was significantly higher than BST-2 in normal breast epithelial cells. However, BST-2 expression between stromal cells (tumors versus normal breast tissues) was not different, indicating that tumor epithelial cells could partly be contributory to elevated BST-2 in tumor tissues. Therefore, BST-2 upregulation may be an important step in a series of changes that tumor cells undergo during transformation.

Intriguingly, we found that the cellular mechanisms responsible for the tumorigenic potential of BST-2 include alterations in cancer cell adhesion, anchorage-independency, migration, and invasion, but not proliferation. In two-dimensional culture, suppression of BST-2 in murine cancer cells had no effect on cell proliferation despite decreased ability of these cells to grow independent of anchor. Although not tested in this study, it is possible that suppression of BST-2 may result in decreased proliferation and increased susceptibility to apoptosis *in vivo*. However, in two-dimensional culture, we found that 4T1 cells but not E0771 cells with suppressed BST-2 expression have higher viability as measured by MTT assay, indicating that cell viability may not be implicated in the role of BST-2 in cancer cell behavior. The attribute of BST-2 that endows it the ability to simultaneously promote so many different malignant processes is yet to be discovered.

It is possible that the expression of BST-2 provides cancer cells a suitable milieu for their growth and spread through the following processes: (i) alteration of cancer cell stiffness enhancing cancer cell adhesion to extracellular matrix and escape from primary tumor [50],[51]; (ii) heightened NF-кB activity and conversion of NF-кB-induced inflammatory stimuli into tumor growth and metastatic signals; (iii) promotion of cancer cells secretion of soluble signaling molecules that potentiate tumor growth and metastasis; and (iv) synthesis of endopeptidases such as matrix metalloproteinases to facilitate degradation of various components of the extracellular matrix, thereby promoting tumorigenesis. Further investigations are required in this respect.

## Conclusions

**Figure 8:**
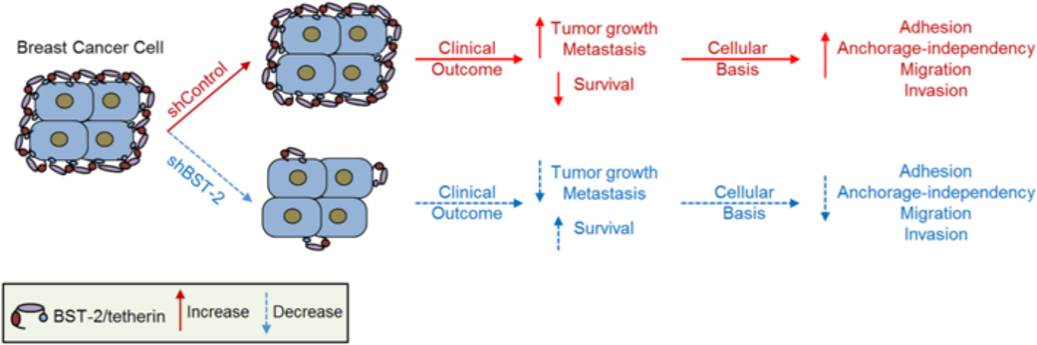
**Schematic portraying the role of cancer cell intrinsic BST-2 in mammary cancer development.** Red arrows portray elevated expression. Blue arrows depict suppressed expression.

The results of this study as summarized in our model (Figure 8) reveal the critical role of BST-2 in the many processes involved in mammary oncogenesis by showing that BST-2 expressed in carcinoma cells is a positive disease modifier and elevated levels of BST-2 predict tumor aggressiveness and host survival. The inability of mammary cancer cells with suppressed BST-2 to efficiently colonize independent of attachment, along with the observed increase in tumor latency in mice implanted with BST-2-suppressed cells suggest that BST-2 action may be early and sustained in the process of mammary tumorigenesis. This report highlights the importance of cell-intrinsic BST-2 in the emergence of neoplasia and malignant progression of breast cancer. Therefore, BST-2 may serve as a biomarker for aggressive breast cancers and as a potential target for the development of new therapeutics for BST-2-dependent cancers.

## Additional files

## Electronic supplementary material

Additional file 1: Figure S1.: BST-2 expression in human breast cancer cell lines. **(A)** Expression of BST-2 mRNA from normal mammary epithelial cells (HMLE), luminal A MCF-7 tumorigenic cells, and claudin-low MDA-MB-231 tumorigenic cells as determined by RT-qPCR. **(B)** BST-2 surface expression from HMLE, MCF-7 and MDA-MB-231 cells as determined by flow cytometry. Numbers in parenthesis correspond to BST-2 expression presented as a percentage. All RT-qPCR data are normalized to GAPDH and presented as fold change over HMLE. Error bars represent standard deviations and significance was taken at *P* <0.01^**^. Experiments were repeated multiple times with similar results. (TIFF 117 KB)

Additional file 2: Figure S2.: BST-2 expressed in mammary cancer cells is suppressed by BST-2-targeting shRNAs. **(A)** Expression of BST-2 mRNA is higher in murine mammary tumor tissues and cells (E0771*luc* and 4T1*luc*) compared to normal mammary gland tissues as determined by RT-qPCR. Following stable transduction of E0771*luc* and 4T1*luc* cells with lentiviruses expressing different BST-2-targeting (sh137 and sh413) and non-targeting (shControl) shRNA, levels of BST-2 **(B and C)** mRNA expression were measured by real-time quantitative PCR, **(D)** surface protein expression was measured by flow cytometry (FACS) and **(E)** total BST-2 protein was measured by Western blot. Numbers correspond to band quantifications. Percent (%) gene expression is calculated as BST-2/GAPDH*100. All RT-qPCR data are normalized to GAPDH and presented as fold change over Normal tissue or shControl cells. Error bars represent standard deviations and significance was taken at *P* <0.01^**^. (TIFF 148 KB)

Additional file 3: Figure S3.: BST-2 downregulation decreases E0771 cell dissemination and growth *in vivo*. **(A)** Knockdown of endogenous BST-2 expression in E0771 cells increases tumor latency. **(B)** Representative images of tumor cells tracked *in vivo* with IVIS imaging system at different time points. Images show higher luciferase bioluminescence in shControl E0771-injected mice compared to sh413-injected mice. **(C)** Representative luciferase bioluminescence accompanied with abdominal and gastrointestinal tract (GI tract) gross images of uninjected (upper panel), shControl-implanted (middle panel), and sh413-implanted mice (lower panel). Arrow heads point to GI tumors. **(D)** Number of secondary tumors in intestine/mesentery plotted as average of all mice. **(E)** Percent incidence of liver and lung metastases. Error bars represent standard deviations and significance was taken at *P* <0.01^**^. (TIFF 1 MB)

Additional file 4: Figure S4.: BST-2 expression in cancer cells predicts host survival. **(A)** Clinical score plot of mice implanted with BST-2-expressing E0771 shControl and BST-2-suppressed sh413 cells. Clinical signs were scored as follows: 0 = no abnormal clinical signs; 1 = ruffled fur but lively; 2 = ruffled fur, activity level slowing, sick; 3 = ruffled fur, eyes squeezed shut, hunched, hardly moving, very sick; 4 = moribund; 5 = dead [23]. **(B)** Representative images of the abdomen and feet of uninjected, shControl, and sh413 C57BL/6 mice implanted with E0771 cells. Arrow points to metastatic ascites (upper-middle panels) and shock (lower-middle panel). **(C)** Kaplan-Meier survival plot of mice implanted with BST-2-expressing shControl and BST-2-suppressed sh413 E0771 cells. Number corresponds to *P* value. Error bars represent standard deviations. Median overall survival (OS) time and the area under the curve (AUC) for each group are shown. (TIFF 336 KB)

Additional file 5: Figure S5.: Figure S5 BST-2 overexpression enhances anchorage-independency, cancer cell migration, and invasion. **(A)** Expression of BST-2 mRNA from MCF-7 cells stably transfected with an empty plasmid (Vector) or with a BST-2-expressing plasmid (WT BST-2) as determined by RT-qPCR. **(B)** Representative images of colonies from a soft agar assay showing anchorage-independent growth of MCF-7 cells. Clones were imaged at 10X. **(C)** Vector-expressing MCF-7 cells form smaller colonies compared to BST-2-expressing MCF-7 cells. Data is presented as percent normalized to Vector-expressing cells. **(D)** Representative images of cell migration by Vector and WT BST-2 expressing cells and Image J quantification of migration events (bars). **(E)** BST-2-expressing and Vector-expressing MCF-7 cells were plated in Matrigel-coated cell inserts and allowed to invade for 24 h. Cells were stained with Giemsa stain. Representative images taken at 20X and Image J quantification of invasion events (bars) are shown. Error bars corresponds to standard deviations. Significance was taken at *P* <0.001^**^ and *P* <0.05^*^. ns = not significant. (TIFF 927 KB)

Additional file 6: Figure S6.: Endogenous BST-2 has no effect on proliferation of mammary cancer cells. **(A and B)** BrdU incorporation assay performed on shControl, sh137, and sh413 E0771 and 4T1 cells respectively. Absorbance was measured at 450 nm using a Tecan Infinite M200 Pro plate reader or cells were imaged using a Zeiss 710 confocal microscope (only for E0771 cells). Images were processed using Image J software. **(C and D)** MTT metabolism assay performed on shControl, sh137, and sh413 E0771 and 4T1 cells to determine cell viability. Absorbance was read at 590 nm using a Tecan Infinite M200 Pro plate reader. Results are expressed as the means ± standard deviations of optical density (OD). BrdU (green), BST-2 (red), and DAPI (blue). Error bars represent standard deviations. Significance was taken at *P* <0.05^*^. ns = not significant. Experiments were repeated multiple times with similar results. (TIFF 474 KB)

Below are the links to the authors’ original submitted files for images.

Authors’ original file for figure 1

Authors’ original file for figure 2

Authors’ original file for figure 3

Authors’ original file for figure 4

Authors’ original file for figure 5

Authors’ original file for figure 6

Authors’ original file for figure 7

Authors’ original file for figure 8

Authors’ original file for figure 9

Authors’ original file for figure 10

Authors’ original file for figure 11

Authors’ original file for figure 12

Authors’ original file for figure 13

Authors’ original file for figure 14

Authors’ original file for figure 15

## Abbreviations

7-AAD: 7-aminoactinomycin D
APC: allophycocyanin
AUC: area under the curve
BRCA: breast-invasive carcinoma
BrdU: bromodeoxyuridine or 5-bromo-2′-deoxyuridine
BSA: bovine serum albumin
BST-2: bone marrow stromal antigen 2
CAFs: cancer-associated fibroblasts
CD317: cluster of differentiation 317
DAPI: 4′,6-diamidino-2-phenylindole
DCIS: ductal carcinoma *in situ*
ECM: extracellular matrix
FACS: fluorescence-activated cell sorting
FBS: fetal bovine serum
FITC: fluorescein isothiocyanate
GEO: Gene Expression Omnibus
H&E: hematoxylin and eosin
HER2: human epidermal growth factor receptor 2
IDC: invasive ductal carcinoma
IgG: immunoglobulin G
MEF: murine embryonic fibroblast
NF-кB: nuclear factor kappa binding
OS: overall survival
PBS: phosphate-buffered saline
PFA: paraformaldehyde
RPMI: Roswell Park Memorial Institute medium
shRNA: short hairpin RNA
TCGA: The Cancer Genome Atlas
TV: tumor volume
