# Supplementary figures and images for: Bone marrow stromal antigen 2 expressed in cancer cells promotes mammary tumor growth and metastasis
Source: Breast Cancer Res. 2014 Dec 13;16:493. doi: 10.1186/s13058-014-0493-8 (PMC4308845; doi:10.1186/s13058-014-0493-8)

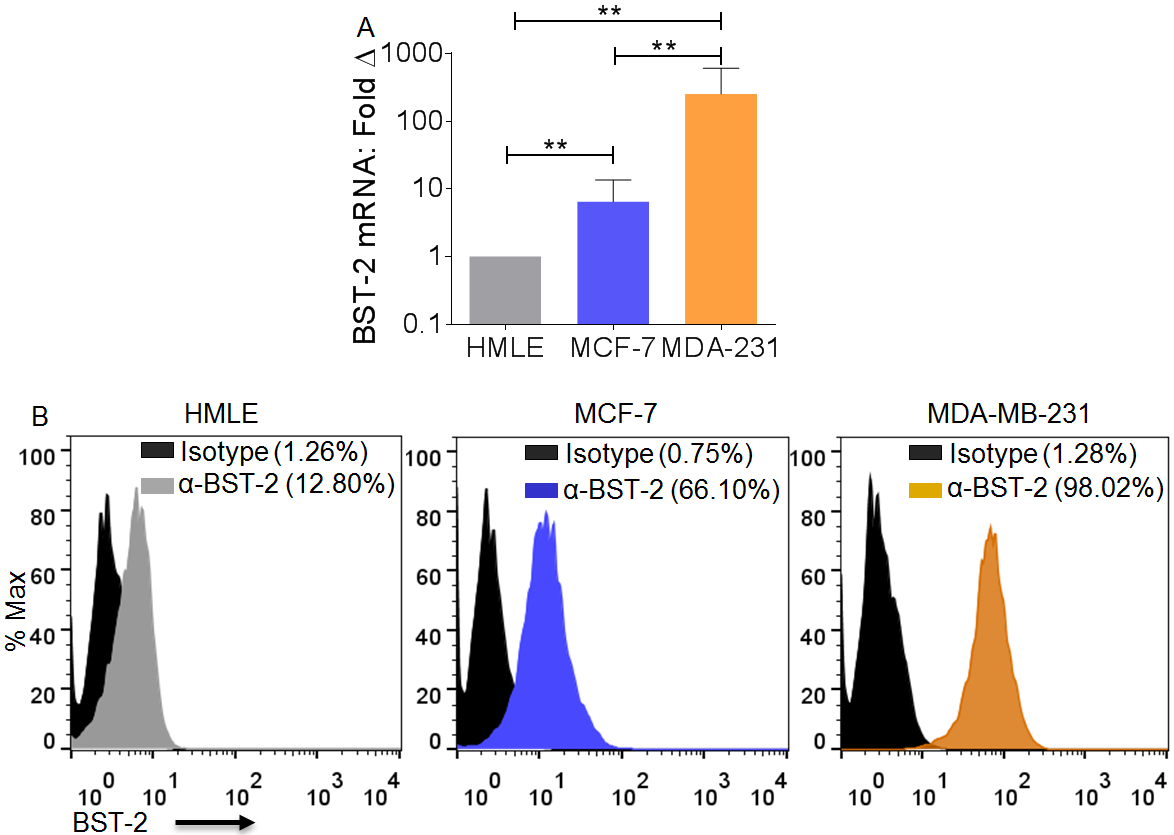

Supplement: Supplementary file 1 — Additional file 1: Figure S1.: BST-2 expression in human breast cancer cell lines. (A) Expression of BST-2 mRNA from normal mammary epithelial cells (HMLE), luminal A MCF-7 tumorigenic cells, and claudin-low MDA-MB-231 tumorigenic cells as determined by RT-qPCR. (B) BST-2 surface expression from HMLE, MCF-7 and MDA-MB-231 cells as determined by flow cytometry. Numbers in parenthesis correspond to BST-2 expression presented as a percentage. All RT-qPCR data are normalized to GAPDH and presented as fold change over HMLE. Error bars represent standard deviations and significance was taken at P <0.01**. Experiments were repeated multiple times with similar results. (TIFF 117 KB) [file 13058_2014_493_MOESM1_ESM.tiff]

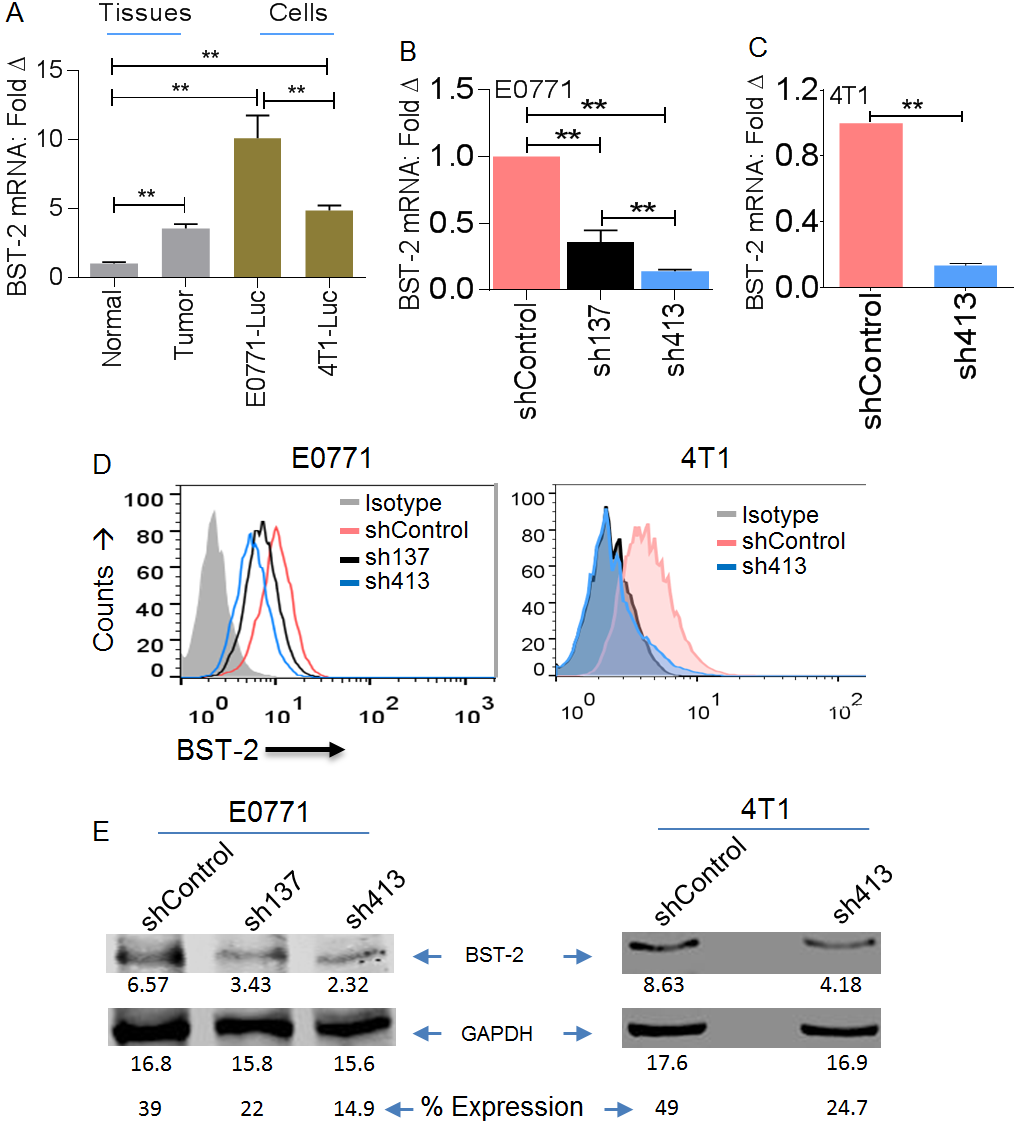

Supplement: Supplementary file 2 — Additional file 2: Figure S2.: BST-2 expressed in mammary cancer cells is suppressed by BST-2-targeting shRNAs. (A) Expression of BST-2 mRNA is higher in murine mammary tumor tissues and cells (E0771luc and 4T1luc) compared to normal mammary gland tissues as determined by RT-qPCR. Following stable transduction of E0771luc and 4T1luc cells with lentiviruses expressing different BST-2-targeting (sh137 and sh413) and non-targeting (shControl) shRNA, levels of BST-2 (B and C) mRNA expression were measured by real-time quantitative PCR, (D) surface protein expression was measured by flow cytometry (FACS) and (E) total BST-2 protein was measured by Western blot. Numbers correspond to band quantifications. Percent (%) gene expression is calculated as BST-2/GAPDH*100. All RT-qPCR data are normalized to GAPDH and presented as fold change over Normal tissue or shControl cells. Error bars represent standard deviations and significance was taken at P <0.01**. (TIFF 148 KB) [file 13058_2014_493_MOESM2_ESM.tiff]

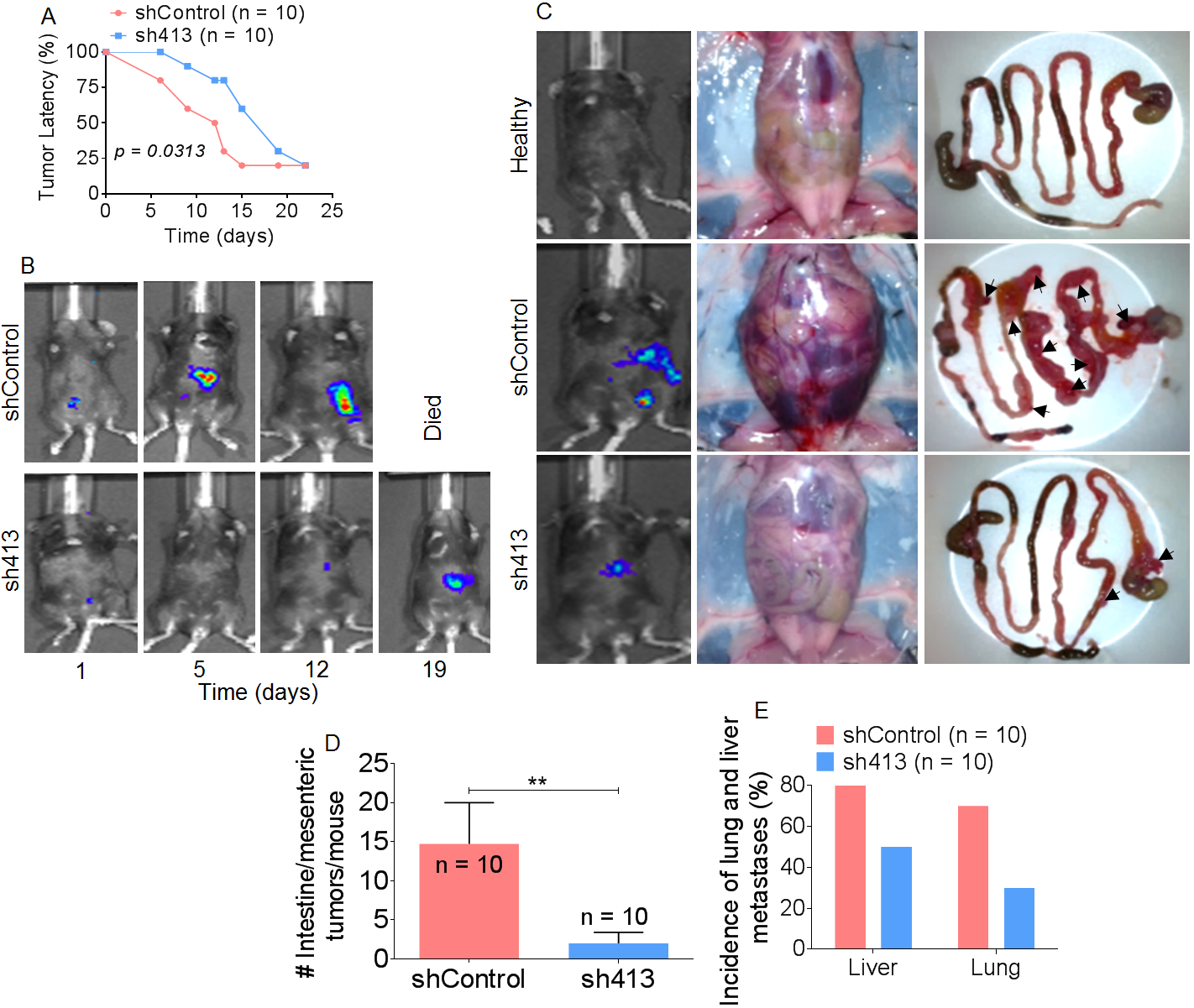

Supplement: Supplementary file 3 — Additional file 3: Figure S3.: BST-2 downregulation decreases E0771 cell dissemination and growth in vivo. (A) Knockdown of endogenous BST-2 expression in E0771 cells increases tumor latency. (B) Representative images of tumor cells tracked in vivo with IVIS imaging system at different time points. Images show higher luciferase bioluminescence in shControl E0771-injected mice compared to sh413-injected mice. (C) Representative luciferase bioluminescence accompanied with abdominal and gastrointestinal tract (GI tract) gross images of uninjected (upper panel), shControl-implanted (middle panel), and sh413-implanted mice (lower panel). Arrow heads point to GI tumors. (D) Number of secondary tumors in intestine/mesentery plotted as average of all mice. (E) Percent incidence of liver and lung metastases. Error bars represent standard deviations and significance was taken at P <0.01**. (TIFF 1 MB) [file 13058_2014_493_MOESM3_ESM.tiff]

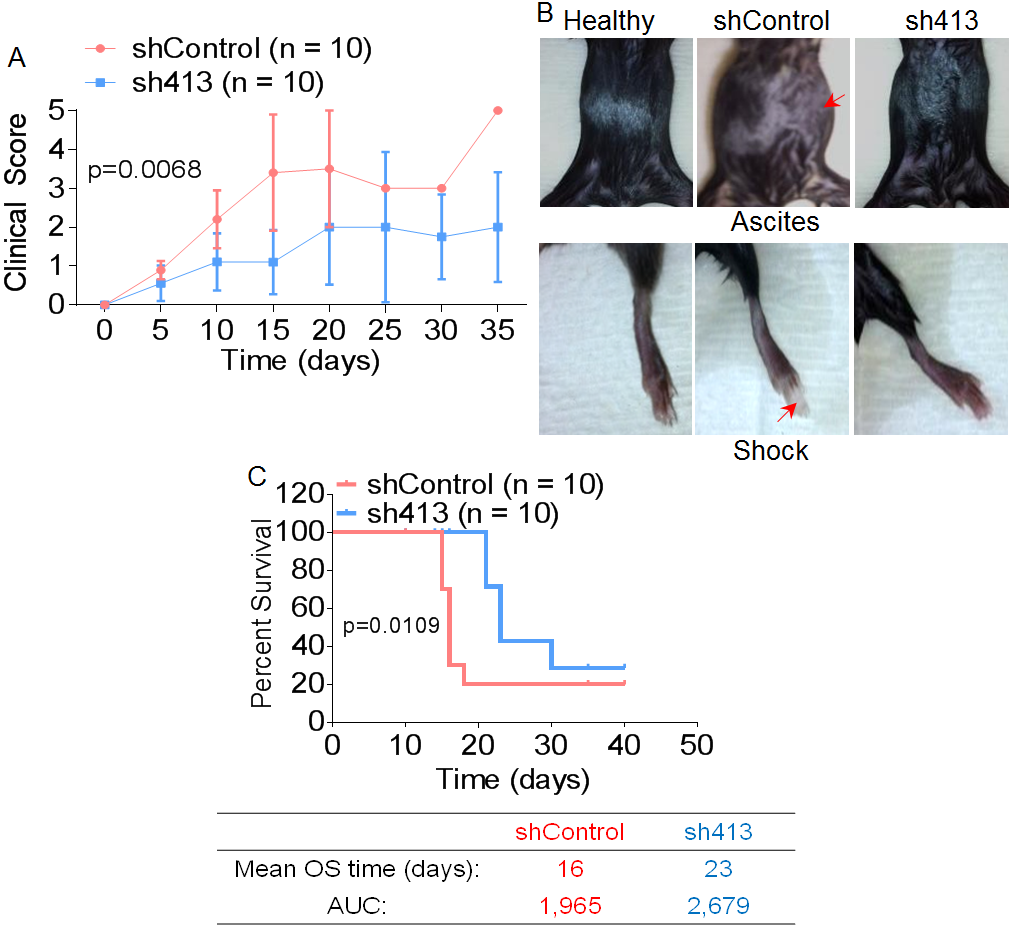

Supplement: Supplementary file 4 — Additional file 4: Figure S4.: BST-2 expression in cancer cells predicts host survival. (A) Clinical score plot of mice implanted with BST-2-expressing E0771 shControl and BST-2-suppressed sh413 cells. Clinical signs were scored as follows: 0 = no abnormal clinical signs; 1 = ruffled fur but lively; 2 = ruffled fur, activity level slowing, sick; 3 = ruffled fur, eyes squeezed shut, hunched, hardly moving, very sick; 4 = moribund; 5 = dead [23]. (B) Representative images of the abdomen and feet of uninjected, shControl, and sh413 C57BL/6 mice implanted with E0771 cells. Arrow points to metastatic ascites (upper-middle panels) and shock (lower-middle panel). (C) Kaplan-Meier survival plot of mice implanted with BST-2-expressing shControl and BST-2-suppressed sh413 E0771 cells. Number corresponds to P value. Error bars represent standard deviations. Median overall survival (OS) time and the area under the curve (AUC) for each group are shown. (TIFF 336 KB) [file 13058_2014_493_MOESM4_ESM.tiff]

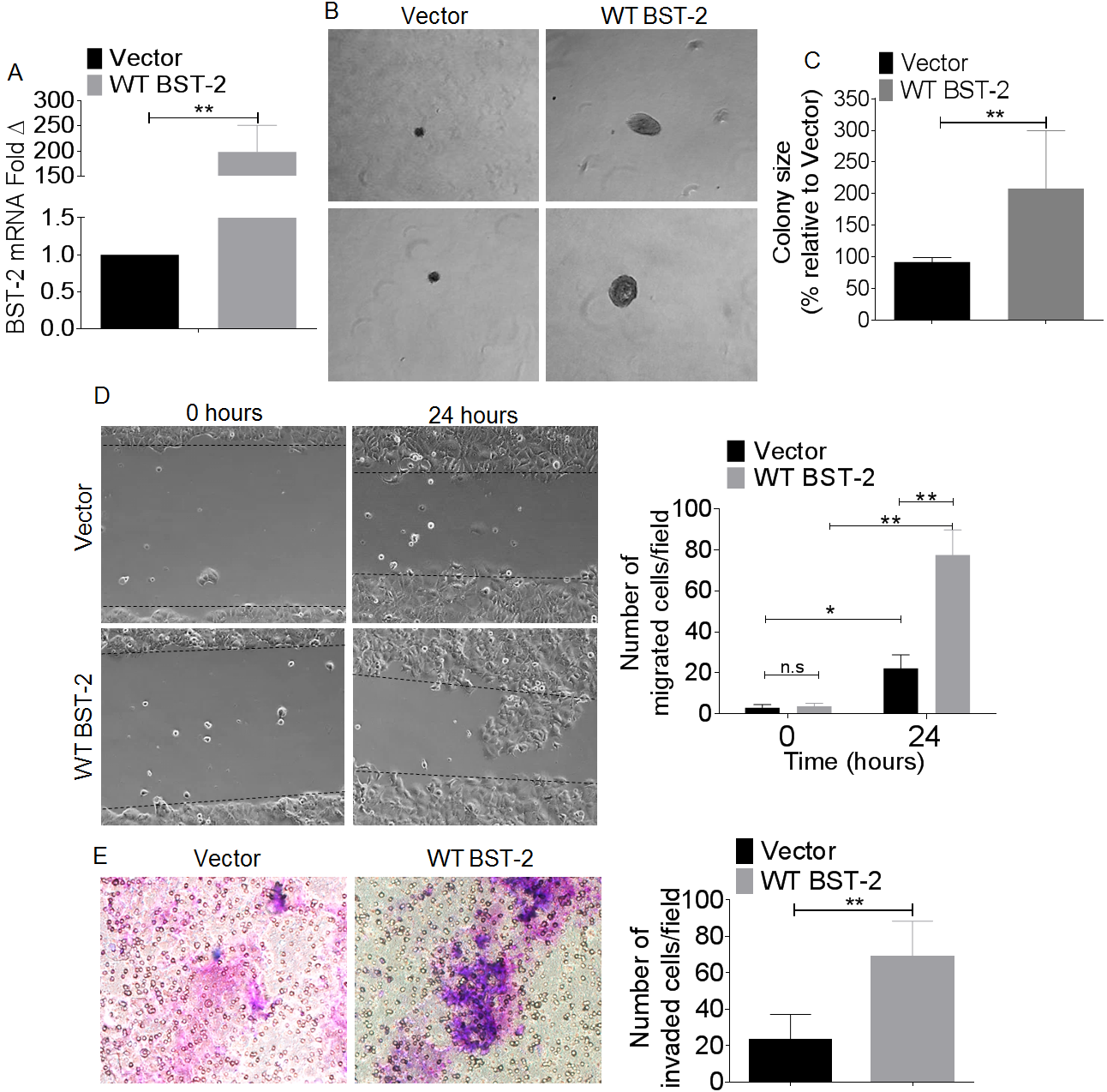

Supplement: Supplementary file 5 — Additional file 5: Figure S5.: Figure S5 BST-2 overexpression enhances anchorage-independency, cancer cell migration, and invasion. (A) Expression of BST-2 mRNA from MCF-7 cells stably transfected with an empty plasmid (Vector) or with a BST-2-expressing plasmid (WT BST-2) as determined by RT-qPCR. (B) Representative images of colonies from a soft agar assay showing anchorage-independent growth of MCF-7 cells. Clones were imaged at 10X. (C) Vector-expressing MCF-7 cells form smaller colonies compared to BST-2-expressing MCF-7 cells. Data is presented as percent normalized to Vector-expressing cells. (D) Representative images of cell migration by Vector and WT BST-2 expressing cells and Image J quantification of migration events (bars). (E) BST-2-expressing and Vector-expressing MCF-7 cells were plated in Matrigel-coated cell inserts and allowed to invade for 24 h. Cells were stained with Giemsa stain. Representative images taken at 20X and Image J quantification of invasion events (bars) are shown. Error bars corresponds to standard deviations. Significance was taken at P <0.001** and P <0.05*. ns = not significant. (TIFF 927 KB) [file 13058_2014_493_MOESM5_ESM.tiff]

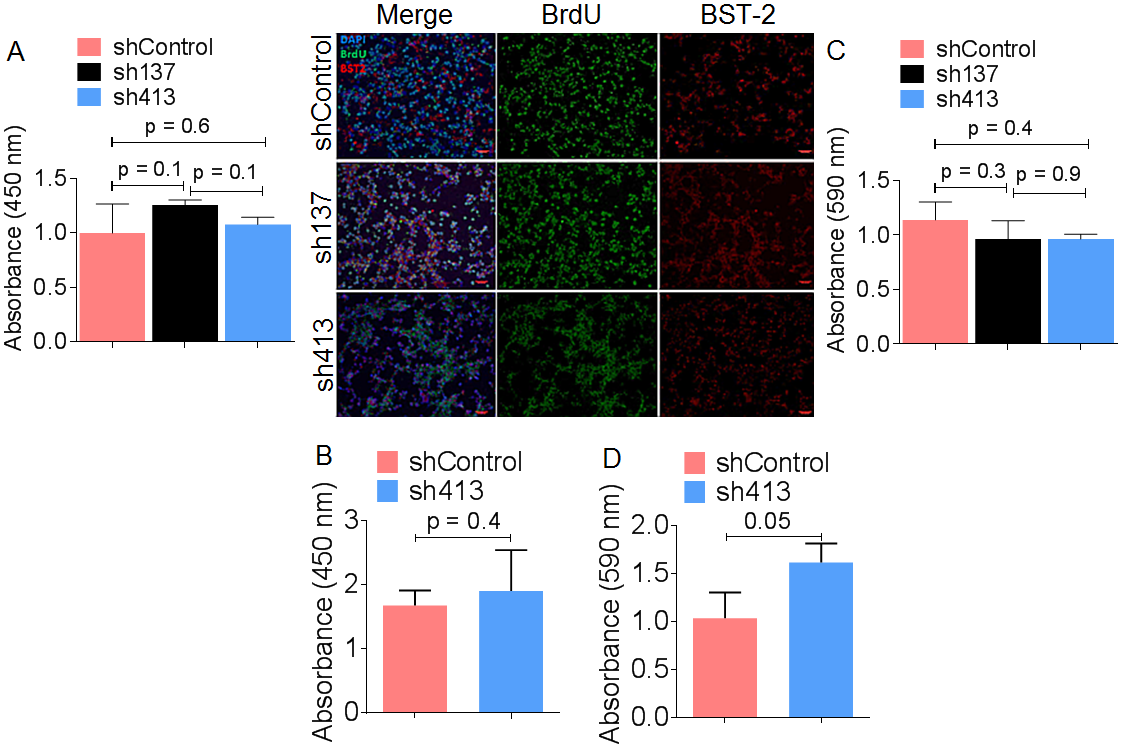

Supplement: Supplementary file 6 — Additional file 6: Figure S6.: Endogenous BST-2 has no effect on proliferation of mammary cancer cells. (A and B) BrdU incorporation assay performed on shControl, sh137, and sh413 E0771 and 4T1 cells respectively. Absorbance was measured at 450 nm using a Tecan Infinite M200 Pro plate reader or cells were imaged using a Zeiss 710 confocal microscope (only for E0771 cells). Images were processed using Image J software. (C and D) MTT metabolism assay performed on shControl, sh137, and sh413 E0771 and 4T1 cells to determine cell viability. Absorbance was read at 590 nm using a Tecan Infinite M200 Pro plate reader. Results are expressed as the means ± standard deviations of optical density (OD). BrdU (green), BST-2 (red), and DAPI (blue). Error bars represent standard deviations. Significance was taken at P <0.05*. ns = not significant. Experiments were repeated multiple times with similar results. (TIFF 474 KB) [file 13058_2014_493_MOESM6_ESM.tiff]

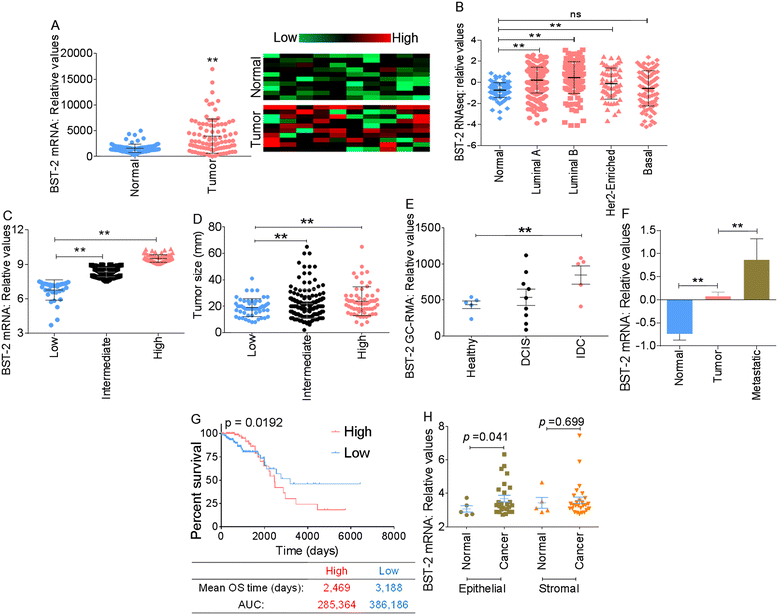

Supplement: Supplementary file 7 — Authors’ original file for figure 1 [file 13058_2014_493_MOESM7_ESM.gif]

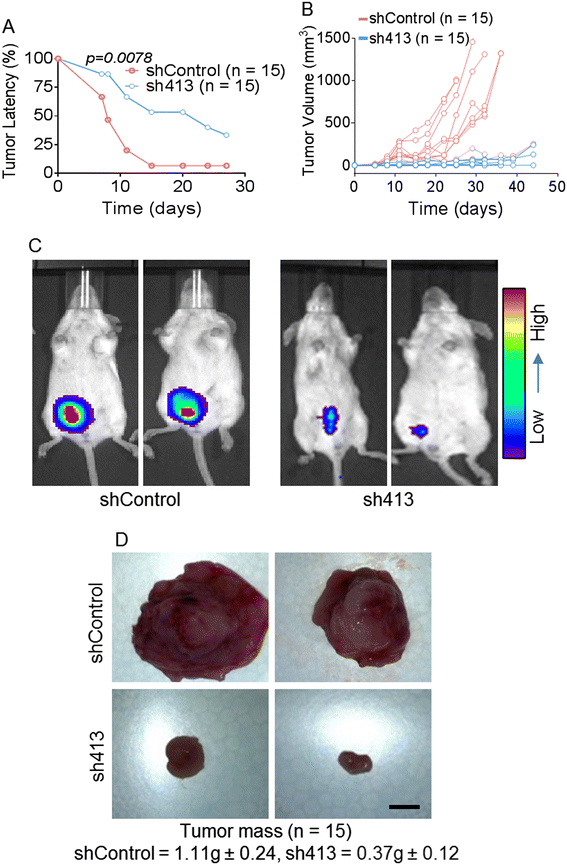

Supplement: Supplementary file 8 — Authors’ original file for figure 2 [file 13058_2014_493_MOESM8_ESM.gif]

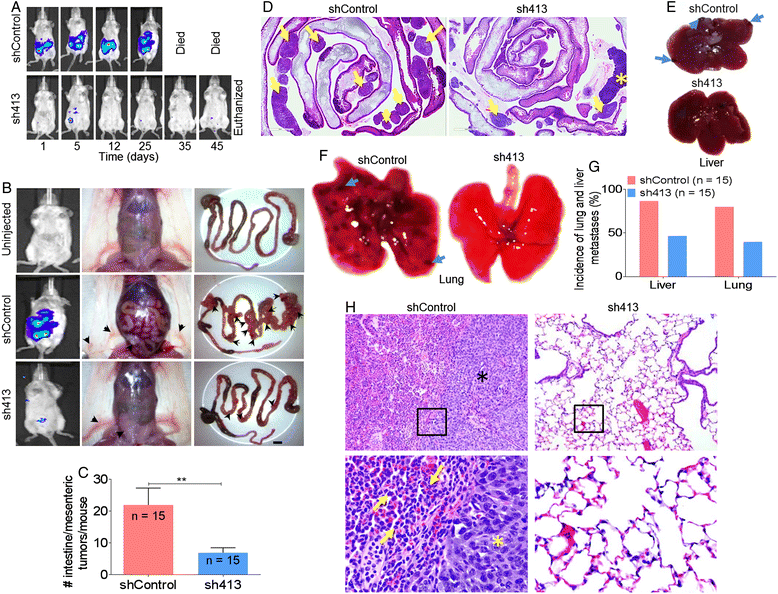

Supplement: Supplementary file 9 — Authors’ original file for figure 3 [file 13058_2014_493_MOESM9_ESM.gif]

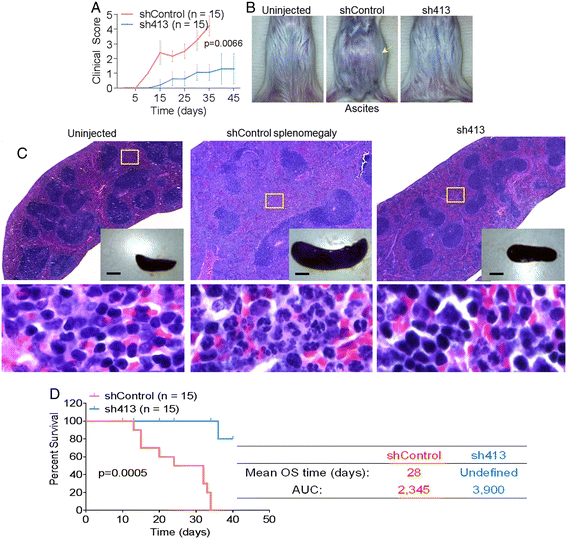

Supplement: Supplementary file 10 — Authors’ original file for figure 4 [file 13058_2014_493_MOESM10_ESM.gif]

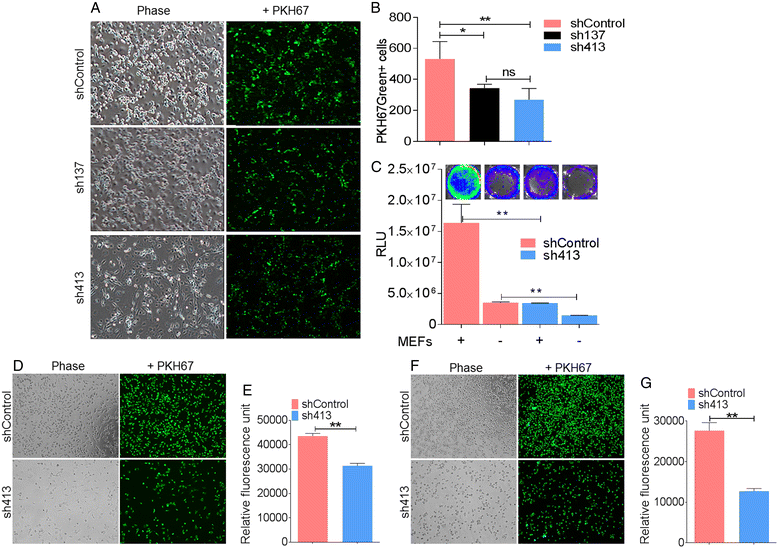

Supplement: Supplementary file 11 — Authors’ original file for figure 5 [file 13058_2014_493_MOESM11_ESM.gif]

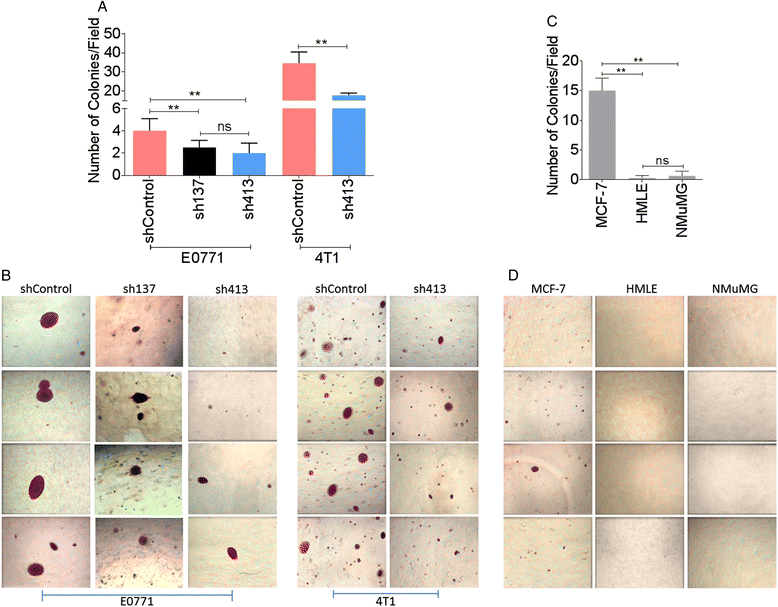

Supplement: Supplementary file 12 — Authors’ original file for figure 6 [file 13058_2014_493_MOESM12_ESM.gif]

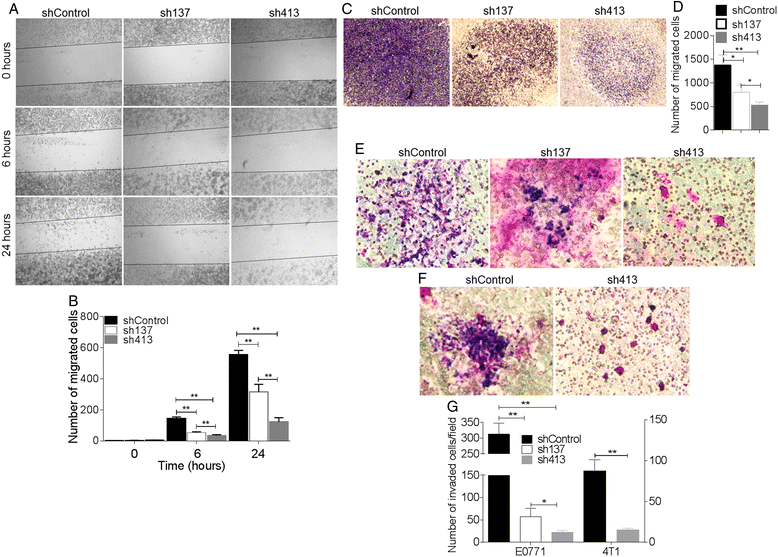

Supplement: Supplementary file 13 — Authors’ original file for figure 7 [file 13058_2014_493_MOESM13_ESM.gif]

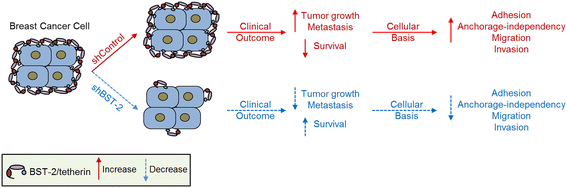

Supplement: Supplementary file 14 — Authors’ original file for figure 8 [file 13058_2014_493_MOESM14_ESM.gif]

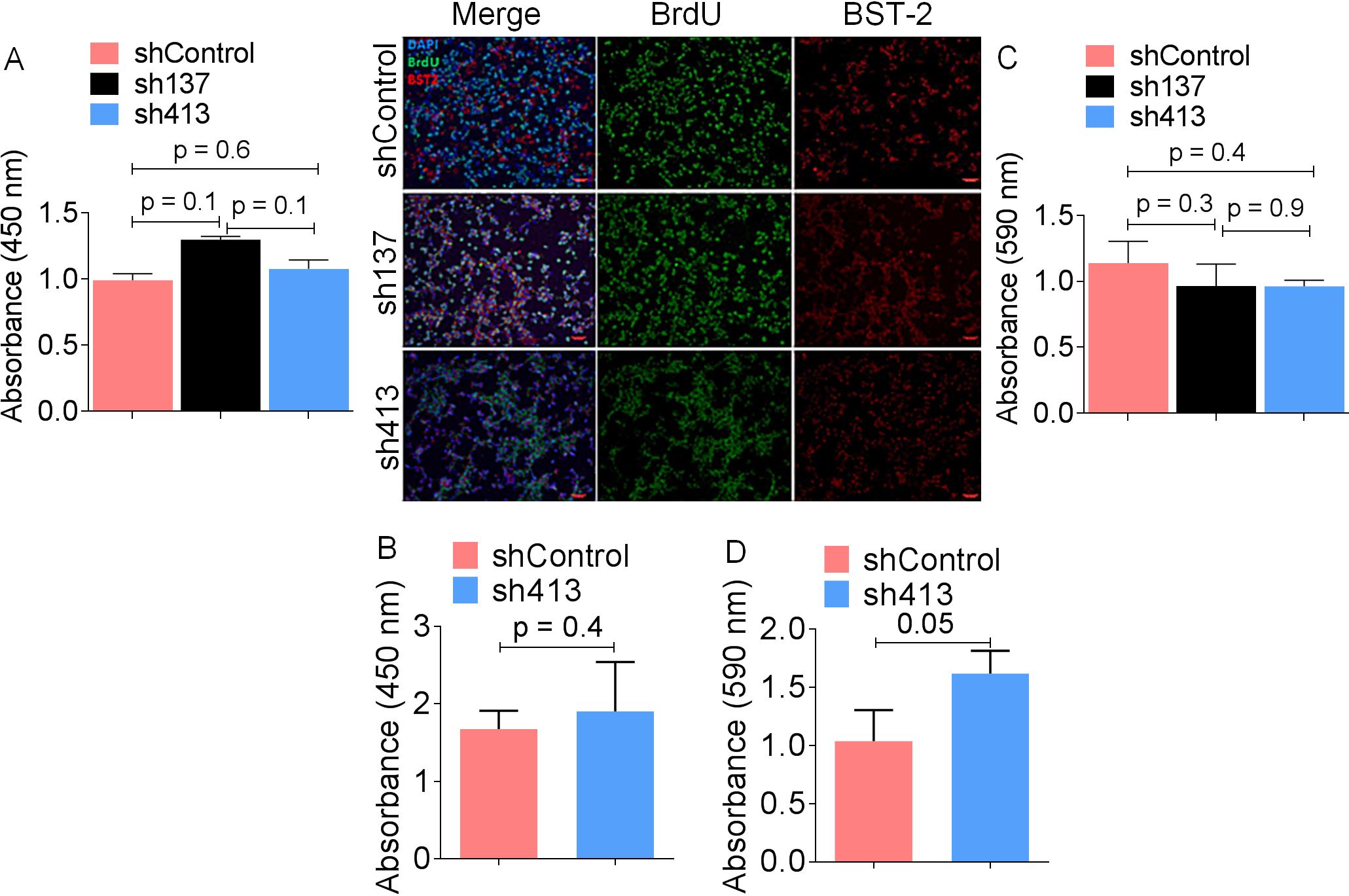

Supplement: Supplementary file 15 — Authors’ original file for figure 9 [file 13058_2014_493_MOESM15_ESM.tiff]

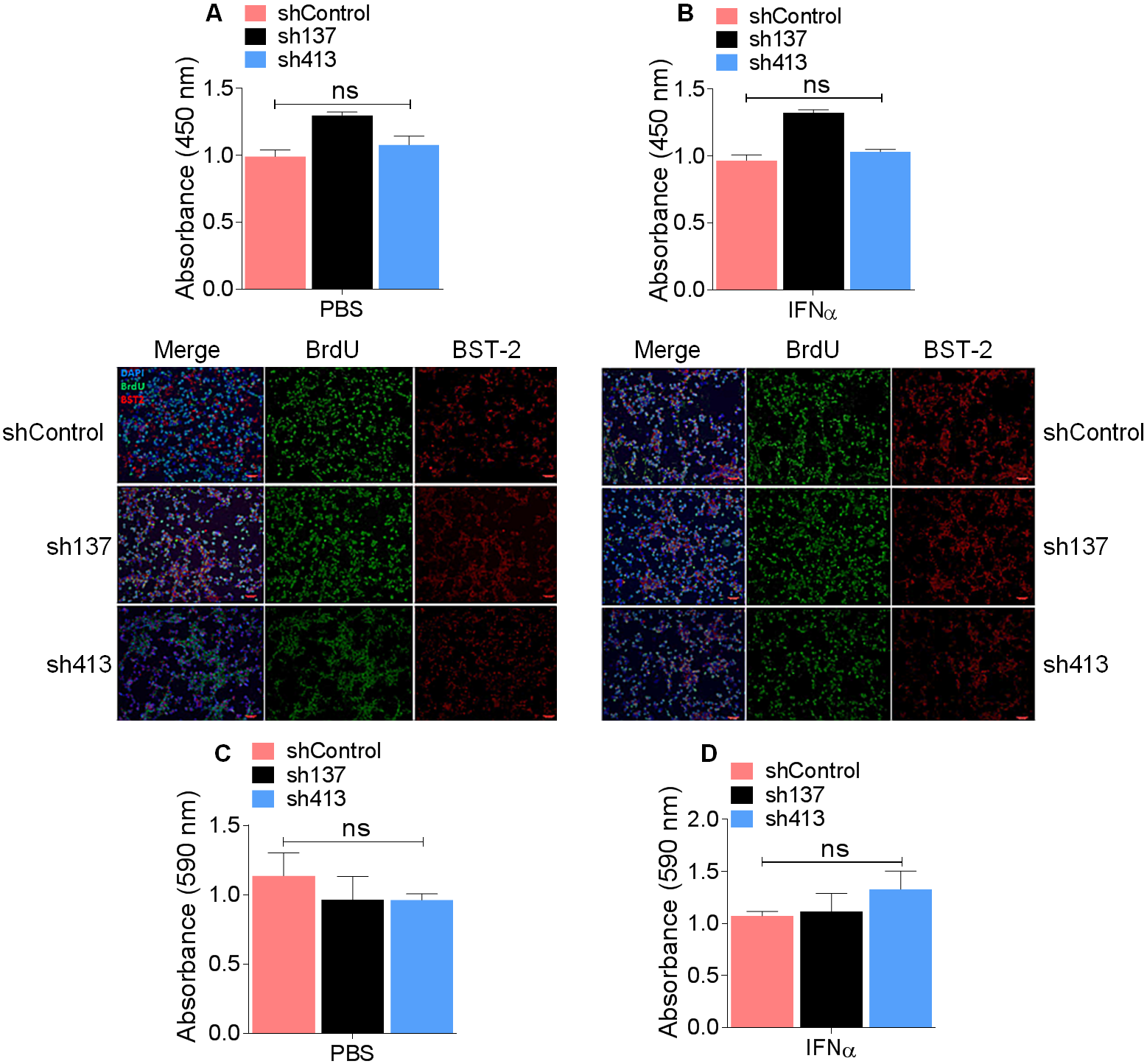

Supplement: Supplementary file 21 — Authors’ original file for figure 15 [file 13058_2014_493_MOESM21_ESM.tiff]
